# Pupillometry in auditory multistability

**DOI:** 10.1371/journal.pone.0252370

**Published:** 2021-06-04

**Authors:** Jan Grenzebach, Thomas G. G. Wegner, Wolfgang Einhäuser, Alexandra Bendixen

**Affiliations:** 1 Cognitive Systems Lab, Institute of Physics, Chemnitz University of Technology, Chemnitz, Germany; 2 Physics of Cognition Group, Institute of Physics, Chemnitz University of Technology, Chemnitz, Germany; Universitatsklinikum Hamburg-Eppendorf, GERMANY

## Abstract

In multistability, a constant stimulus induces alternating perceptual interpretations. For many forms of visual multistability, the transition from one interpretation to another (“perceptual switch”) is accompanied by a dilation of the pupil. Here we ask whether the same holds for auditory multistability, specifically auditory streaming. Two tones were played in alternation, yielding four distinct interpretations: the tones can be perceived as one integrated percept (single sound source), or as segregated with either tone or both tones in the foreground. We found that the pupil dilates significantly around the time a perceptual switch is reported (“multistable condition”). When participants instead responded to actual stimulus changes that closely mimicked the multistable perceptual experience (“replay condition”), the pupil dilated more around such responses than in multistability. This still held when data were corrected for the pupil response to the stimulus change as such. Hence, active responses to an exogeneous stimulus change trigger a stronger or temporally more confined pupil dilation than responses to an endogenous perceptual switch. In another condition, participants randomly pressed the buttons used for reporting multistability. In Study 1, this “random condition” failed to sufficiently mimic the temporal pattern of multistability. By adapting the instructions, in Study 2 we obtained a response pattern more similar to the multistable condition. In this case, the pupil dilated significantly around the random button presses. Albeit numerically smaller, this pupil response was not significantly different from the multistable condition. While there are several possible explanations–related, e.g., to the decision to respond–this underlines the difficulty to isolate a purely perceptual effect in multistability. Our data extend previous findings from visual to auditory multistability. They highlight methodological challenges in interpreting such data and suggest possible approaches to meet them, including a novel stimulus to simulate the experience of perceptual switches in auditory streaming.

## Introduction

Sensory information often contains ambiguities, such that the exact same input can be interpreted in different ways [1]. In most cases, we do not notice the ambiguity because our perceptual system chooses the most probable or plausible interpretation, which then dominates perception [2]. However, when two or more alternative interpretations are about equally probable, the phenomenon of perceptual multistability is observed [3]: Despite physically constant stimulation, perception alternates between the different interpretations (*percepts)*. Changes between percepts are referred to as perceptual *switch*. Following Leopold and Logothetis [3], perceptual switching during multistability can be characterized by its *randomness* (with respect to percept duration, as well as with respect to the percept sequence in case of more than two alternatives), its *exclusivity* (only one percept is experienced at any given moment) and its *inevitability* (switching cannot be avoided).

Multistability occurs in many sensory modalities [4] and has been studied in great detail in vision (e.g., [5]) and audition (e.g., [6]). Similar computational principles [7–12] and brain areas [13–16] have been implicated for various forms of multistability, suggesting that they may share common mechanisms. If such mechanisms are shared across modalities, physiological responses to visual and auditory multistability should be similar. Here we consider a psychophysiological indicator that might accompany perceptual switching in both modalities alike: the constriction and dilation of the pupil. Einhäuser and colleagues [17] showed that momentary pupil dilation accompanies perceptual switches in three forms of *visual* bistability (moving plaid, structure-from-motion, and Necker cube). They found numerically consistent results for the *auditory* modality (auditory streaming), which failed to reach significance. Pupil dilations for visual multistability were confirmed and further confined by Hupé, Lamirel, and Lorenceau [18]. A more comprehensive test of pupil dilation effects in auditory multistability has not been conducted so far [19]. In the current study, we examine pupillary dynamics during auditory multistability. Finding dynamics similar to previous results in vision [17, 18] would speak in favor of partly overlapping mechanisms for multistability both modalities.

Of the many cognitive factors that affect pupil size (see [20–22] for recent reviews), several might be linked to the pupil-size change around perceptual switches. The pupil may dilate in response to a perceptual switch because arousal mediates perceptual switching [23] and pupil dilation [24], because switches are rare events, which trigger pupil dilation [25, 26], because inferring and changing the current perceptual state involves a decision process [27, 28], or because the switch changes the level of uncertainty [29]. Most of these concepts are rather broad, to some extent interwoven, and details with respect to pupil size often depend on their precise operationalization. Even though we do not aim at disentangling the unique contributions of these factors in the present study, it is particularly critical to devise conditions that control these factors as carefully as possible when studying pupil size in multistability.

We conducted two experiments combining a standard paradigm of auditory multistability, the auditory streaming paradigm, with pupillometry. The auditory streaming paradigm involves presenting a sequence of interleaved ‘A’ and ‘B’ tones (ABABABAB…) that differ from each other in frequency [30, 31]. This stimulus configuration can be interpreted as originating from one sound source producing tones of two different frequencies (the *integrated* percept) or from two sound sources with distinct frequencies (the *segregated* percept). In the case of perceptual segregation, we additionally asked which of the sound sources–both sources, only the one producing the high sounds, or only the one producing the low sounds–are perceived in the foreground [32]. The multistable nature of perception of various versions of this auditory stimulus has been well characterized [33–35].

For observing a perceptual switch during unchanged physical stimulation, we have to rely on the participant’s overt report. Making the switch accessible to report requires introspection and metacognitive evaluation [5], whereas the report as such involves decision-making, motor planning, and motor execution [36]. These processes evoke pupillary components themselves. Therefore, a pupillary response at the time of an overtly reported perceptual switch might be contaminated or overshadowed by the report processes [37], rather than being driven by the perceptual switch itself. To control for such potential confounds, we applied control conditions that involve pressing a report-button (like in the multistable condition) to compensate for the sensitivity of the pupil to motor actions [38, 39]. Specifically, in the *random* control condition, the participants press buttons at randomly chosen time points, without any temporal relation to perceptual switching [18, 40]. This gives a direct control for the *multistable* experimental condition, in which participants press buttons contingent upon their perceptual changes during ambiguous input. In a further control condition, the *replay* condition, participants likewise press buttons in response to perceptual changes, but this time the changes are exogenously elicited by stimulus changes, rather than caused by endogenous re-interpretations of physically unchanging input (see [41] for a detailed discussion of replay conditions in visual multistability). The stimulus changes constitute a direct replay of the participant’s behavioral switching dynamics in the multistable condition, transformed into disambiguated versions of the stimulus [17].

Study 1 involves these three conditions (multistable, replay, and random). In each of the conditions, we inspect the average pupillary response around the button press. If the pupil dilates specifically in response to the perceptual switch during multistable perception of ambiguous auditory input, we expect the following result pattern:

- Hypothesis 1: The pupil dilates in response to, or slightly preceding, a perceptual switch in the multistable condition [17].
- Hypothesis 2 (motor control): The pupil dilation to a perceptual switch (reported by button press) in the multistable condition is larger than the pupil dilation to a button press without perceptual switch in the random condition [18].
- Hypothesis 3 (physical change control): The pupil dilation to an endogenous perceptual switch in the multistable condition is larger than to an exogenous perceptual switch in the replay condition.

## Study 1: Materials and method

### Participants

20 healthy volunteers (age [mean ± standard deviation]: 26.4 ± 3.33 years; 10 male, 10 female; 3 left-handed, 17 right-handed), who were recruited from the university community, gave their written consent to take part in this study after being informed about the study, yet staying naïve regarding the hypotheses. Data collection for two additional volunteers was commenced but had to be cancelled for technical reasons. Participants received monetary compensation or course credit for the ca. 1.5h long study. All procedures conformed to the principles laid out in the Declaration of Helsinki and were determined by the applicable body (*Ethikkommission der Fakultät für Human- und Sozialwissenschaften*, *TU Chemnitz*) not to require in-depth ethics evaluation (case no. V-219-15-AB-Modalität-14082017).

### Setup

We conducted the experiment in a testing chamber (“Type 120a double-walled”, IAC Acoustics, UK), which is a dark room (no light source other than the monitor) with sound-absorbing walls (ambient noise level ≤ 16.9 dB(A)). A 24”-display (“VG248QE”, Asus; Taiwan) was positioned outside of the chamber and was visible through a multi-layer glass window. We measured the pupil diameter and gaze position of the right eye at 1 000 Hz sampling rate with the “Eyelink-1000” camera/infrared-system (SR Research, Canada) positioned inside the chamber in Desktop mount configuration. The participant rested with the head in a chin and forehead rest (for optimal, stable viewing point of the eye-tracking camera).

Participants gave manual responses via a four-button box, “Blackbox” (The Black Box ToolKit Ltd., UK), in front of them. The button box was connected over USB with the “Display PC” used for presenting the stimuli and over the parallel port with the PC used for recording the eye data (Eyelink’s “Host PC”), providing a high degree of synchrony with the pupil signal. The four buttons were positioned on the four edges of an isosceles trapezoid with the shorter “base side” towards the participant. The participants were seated with their fingers resting on the four buttons. They were instructed to use the index fingers of both hands for the two nearer buttons on the button box and the middle fingers to actuate the two farther buttons.

The auditory stimuli were presented binaurally via “HD25-1 II” headphones (70 Ohm, Sennheiser, Germany) over the soundcard “Sound Blaster X-Fi Titanium HD, SB1270” (Creative, USA) inside a high-performance stimulus-PC running Arch Linux, Matlab R2015b (Mathworks, USA) and “Psychophysics Toolbox” [42] with eyelink-toolbox-extension [43]. The Display PC was used for experiment control (e.g., instructions; eyelink-control), auditory and visual stimulus presentation, and behavioral response acquisition (button presses).

The ambient noise level and experimental sound levels were externally verified with “Type 2270” hand-held sound level meter and “Type 4101-A” binaural microphones (both by Bruel & Kjaer, Denmark). The timing of the sounds was externally verified with an oscilloscope and recorded internally with a jitter of sound start time of less than 25 μs (observed over 1 200 played sounds). Sound start and end were communicated from the Display PC to the Eyelink Host PC over the parallel port for redundant recording.

### Auditory stimuli

The auditory sequence was a combination of two sinusoidal sounds (A and B; duration 230 ms each), which were played alternatingly with a 20 ms break in between: sound A, 20 ms silence, sound B, 20 ms silence, and so on. This resulted in a stimulation rate of four sounds per second. The same stimuli were presented to both ears. Each sound was gated with 5-ms raised-cosine onset and offset ramps to avoid acoustic artifacts in the headphone evoked by the sudden deflection of the sound membrane.

The frequency trajectories of A and B as well as their sound levels varied according to the condition. The multistable condition employed constant-frequency sounds (A: 400 Hz, B: 712 Hz, i.e., 10 semitones apart) at a level of 75 dB(A). At the end of each block of the multistable condition, the sound level of either A or B was lowered to 50 dB(A) in some segments to verify participants’ responses (see *Procedure*). The random condition employed the same sounds as the multistable condition without the response verification segments in the end.

**Fig 1:**
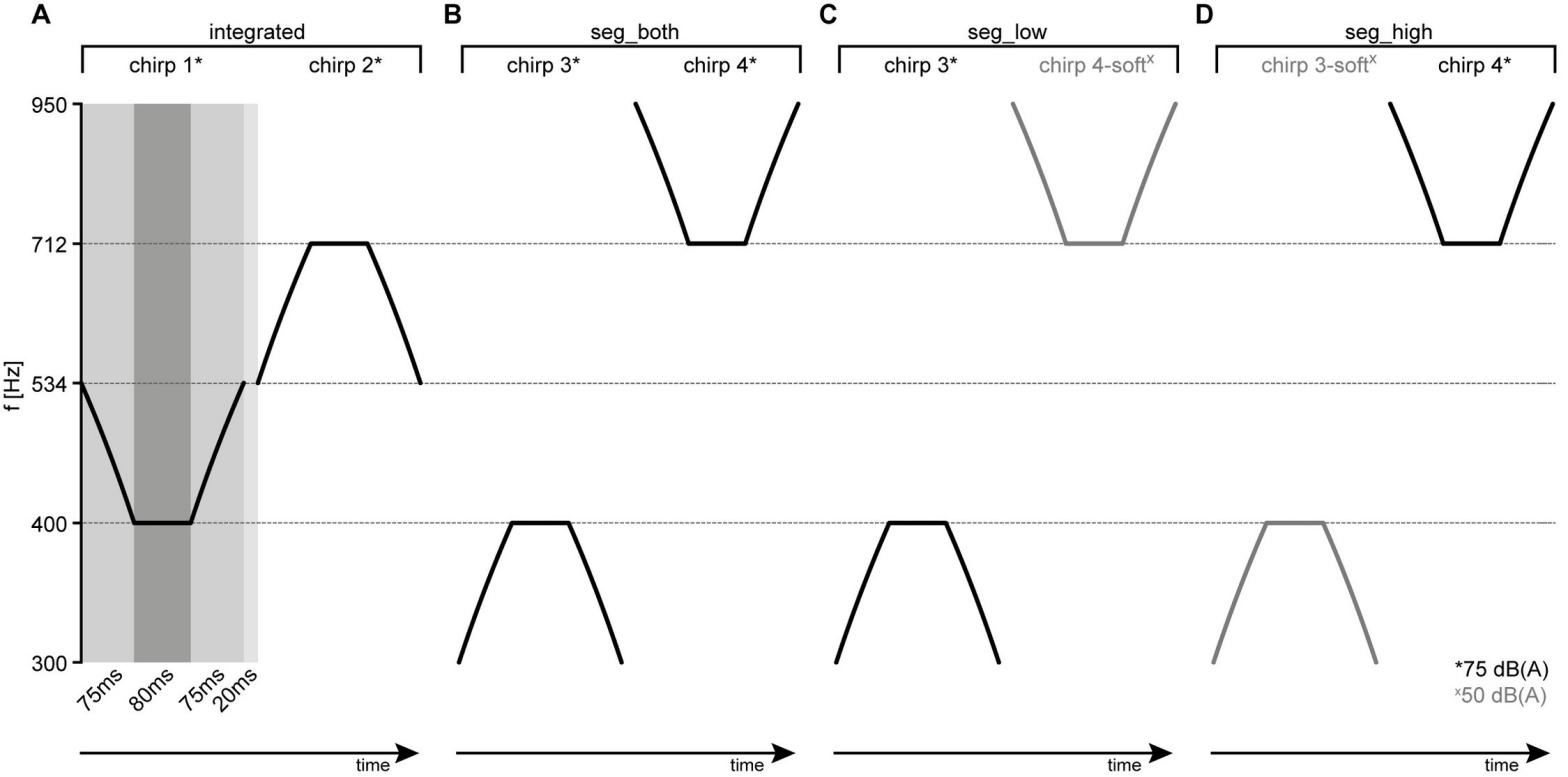
Sound design for the replay condition. **A**) Sounds mimicking the integrated percept (“int”) consisting of repeating chirps 1 [sound A] and 2 [sound B]; with the drift of frequency inside the sound. **B-D**) Sounds mimicking the segregated percepts. **B**) “seg_both” (chirps 3 [tone A] and 4 [tone B]); **C**) “seg_low” (chirps 3 [tone A] and 4-soft [tone B]) **D**) “seg_high” (chirps 3-soft [tone A] and 4 [tone B]). Note that the spacing is uniform on a log scale (i.e., the frequencies represent steps of 5 semitones), while the sweep itself is linear in frequency, hence the slight curvature in log space. Chirps with reduced level (50dB(A)) are depicted in gray (chirps 3-soft and 4-soft).

The replay condition employed changing-frequency sounds (chirp sounds) with a middle frequency of 400 Hz (A) and 712 Hz (B). The chirp sounds consisted of three parts (see Fig 1A): First, a ramp (75 ms) starting with an “initial frequency”, which was changed linearly to the second (middle) frequency which remained constant for 80 ms, and third, a ramp (75 ms) where the middle frequency was linearly changed back to the initial frequency. For replay stimuli designed to mimic the integrated percept (chirps 1 and 2, see Fig 1A), the initial frequency was the geometric mean between the two middle frequencies (534 Hz), such that the A and B tones both started at 534 Hz, changed towards 400 Hz (A) or 712 Hz (B), and changed back to 534 Hz. For replay stimuli designed to mimic the segregated percept (chirps 3 and 4, see Fig 1B), the initial frequency was 300 Hz for A tones and 950 Hz for B tones (i.e., 5 semitones below 400 Hz and 5 semitones above 712 Hz, respectively). Chirps 1 to 4 were presented at a level of 75 dB(A). For additionally mimicking foreground-background perception in the segregated case, chirps 3-soft and 4-soft (see Fig 1C and 1D) were identical to chirps 3 and 4 but presented with lower levels of 50 dB(A). Chirps 3 and 4-soft were thus combined to mimic segregated-foregroundA, chirps 3-soft and 4 were combined to mimic segregated-foregroundB, and chirps 3 and 4 were combined to mimic segregated-both in the foreground.

### Visual stimuli

During each type of auditory stimulation, the display showed a light gray fixation square (luminance: 88.1 cd/m^2^; edge length: 3.1 degrees of visual angle) in the center of a black screen. Participants were asked to direct their gaze inside the fixation square during all times.

### Procedure

The experiment was divided into eight blocks: three blocks of the multistable condition (block numbers 2, 4 & 6; each 5 min), each of which was directly followed by a replay block (block numbers 3, 5 & 7; each 4 min). The first and the last block of the experiment belonged to the random condition (block number 1 & 8; each 4 min). Individual training blocks were administered before the first appearance of the multistable (2) and the replay (3) blocks, respectively.

Before each block, the eye-tracker was calibrated with a 9-point-calibration (covering the center part of the display). Any new block type was (re-)introduced by a written instruction on paper. Instructions (e.g., mapping of the buttons) were reiterated by the experimenter after participants had read the paper instruction, and were summarized on the display before the block start. The start of each block was in control of the participant. After each block, the door of the chamber was opened, and the participant had the chance to pause.

#### Multistable condition

**Fig 2:**
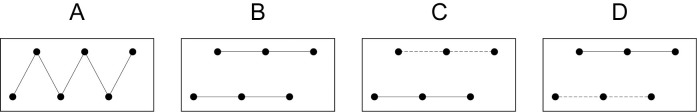
Perceptual alternatives in the multistable condition. Original depiction taken from the paper instruction; dots: single sounds; lines: perceptual organization (solid: foreground; dashed: background); **A**) Integrated percept (“int”); **B**) Segregated percept, both streams in the foreground (“seg_both”); **C**) Segregated percept, low stream in the foreground (“seg_low”); **D**) Segregated percept, high stream in the foreground (“seg_high”).

For a duration of 4 min, the constant-frequency sounds A and B were presented at a level of 75 dB(A), and participants were asked to indicate their subjective perception continuously. The instruction emphasized the subjective nature of perception and introduced the possible percepts and the corresponding buttons as follows: The integrated percept (abbreviation: “int”) was defined as both sounds forming one continuous stream (ABAB…, see panel A of Fig 2). The segregated percept was defined as the two sounds forming two separate streams, none of which is perceived as dominating (A_A_… and B_B_…, “seg_both”, panel B). The segregated percept with the low stream in the foreground (A_A_…, “seg_low”, panel C) and the segregated percept with the high stream in the foreground (B_B_…, “seg_high”, panel D) were defined as the two sounds forming two separate streams, of which either the low or the high stream is perceived as dominant. The percepts were illustrated with the pictograms shown in Fig 2.

To rule out any effects of manual difficulty or finger preference, the assignment of the four perceptual alternatives to the four response buttons varied between participants. The “int” and “seg_both” percepts were always paired on one side (but switching on the lower/higher buttons) of the button box, and the “seg_low” and “seg_high” percepts were paired on the other side of the button box, with “seg_low” always being on the lower button for reasons of feature-response compatibility. This results in four unique combinations of button-response mappings, which were counterbalanced across participants.

Participants were instructed to press and hold the button as soon and as long as they experienced the respective percept. They were asked to release the button when a perceptual switch occurred and to press the button corresponding to the new percept as soon as they had identified the percept. If they could not categorize their momentary perception into one of the four alternatives or if they were confused, they were asked to release all buttons until a percept mapping to one of the alternatives would return. It was highlighted that perception of such sequences is a highly subjective process, and that they should not attempt to actively manipulate the process.

After the 4-min ambiguous part of the multistable condition, a 1-minute response verification part was administered. In this part, segments with reduced level (50 dB(A)) of either one of the tones were presented in random succession with segments of full level (75 dB(A)) for both tones. The three types of segments alternated randomly and continuously with a varying duration of 5 to 9 s for each segment. Lowering the sound level of one tone type (A or B) was expected to lead to a segregated percept with the other tone type (B or A, respectively) in the foreground, and participants’ responses could thus be checked for whether the correct button-response-mapping was employed. The recognition of tones A and B and their mapping to the correct button (A/B in foreground) was practiced in training blocks before the first block of the multistable condition (i.e., before block 2 of the experiment).

The purpose of the training blocks for the multistable condition was to familiarize the participants with their multistable perception of the ambiguous sequence and to help them memorize the mapping of the buttons to the four different perceptual alternatives. The first training block (2 min duration) focused on training to distinguish and classify the low (A) and high (B) tones. For this purpose, segments with only one of the tone types present (i.e., only A or only B tones) were presented in alternation with ambiguous segments (both A and B at 75 dB(A)) with a varying length of 5 to 9 s for each segment. The second training block (3 min duration) was identical to the response verification segments of the experimental blocks: the “background” tone type was no longer absent but presented with reduced level. Again, three types of segments (reduced level of A, reduced level of B, or full level of both tones) alternated randomly with a varying length of 5 to 9 s for each segment. For online evaluation, training hit rate was based on segments with reduced level of A or B tones only (because any response would have been correct for segments with full level of both tones, due to their ambiguity). Hit rate was defined as the summed time of exclusively pressing the correctly associated button (“seg_high” for reduced level of A, and “seg_low” for reduced level of B) divided by the total presentation time of segments with one tone type lowered in level. A maximum of 3 training blocks of each type were administered. If hit rate reached 80% earlier, further training blocks were waived.

#### Replay condition

Each replay block played back, in a disambiguated manner, the participant’s own sequence of percepts in the ambiguous part of the immediately preceding multistable block. Disambiguation was implemented via chirp sounds (see *Auditory Stimuli*). Only valid button-press events (i.e., phases with the exclusive press of a single button, indicating a unique percept) of the multistable condition were replayed. No- or double-button presses were excluded; their cumulative times were distributed equally to the valid phases such that the overall duration of 4 min was kept. The order of the segments was shuffled for the replay relative to the multistable condition. The replay instruction emphasized that in the current task, the segments could be identified correctly. Participants were instructed to press and hold the button as soon and as long as they were presented with the replay segment corresponding to that button.

Identification of each replayed percept and mapping to the correct button was practiced in training blocks before the first block of the replay condition (i.e., before block 3 of the experiment). For a duration of 2 min, the four replay segments alternated randomly with a varying length of 5 to 9 s for each segment. Training hit rate was defined as the summed time of exclusively pressing the button that was correctly associated with the current replay segment, divided by the total presentation time of the respective replay segment type. A maximum of 3 training blocks was administered. If hit rate reached 80% (separately for each of the four types of replay segments) earlier, further training blocks were waived.

Before each training and experimental block, the four different replay segments were presented for 13 s each, along with a reminder of the corresponding label and button.

#### Random condition

In the first and the last block of the experiment, participants were asked to press the four buttons randomly while listening to the same sequences as in the ambiguous part of the multistable condition for a duration of 4 min. During the first block, participants had not received any instructions about the stimulus or the possible ways of perceiving it, whilst in the last block, they had such knowledge based on their experience with the multistable condition. The instruction for the random condition asked participants to always hold only one button for several seconds before switching to one of the remaining three buttons, and to refrain from any planned order in the button sequence, as well as from any rhythm in the timing of presses and releases. The auditory sequence was explained to be irrelevant for the task.

#### Aggregation

All dependent variables (pupil dilation, button-press duration, response verification and replay performance) were averaged in the following manner: All observations made for a participant and condition were aggregated from the different blocks, and then averaged within the participant. The within-participant averages were averaged across participants separately for each condition.

#### Response verification performance (multistable condition)

Ambiguous segments were discarded from hit-rate analysis in the multistable condition because the responses could not meaningfully be classified as correct or incorrect. Further, unlike for online evaluation during training (see above), all segments with reduced level of one tone type (low or high) in which participants reported to perceive “int” or “seg_both” were discarded because participants’ post-experimental reports suggested that these responses corresponded validly to their subjective perception rather than being misclassifications. Thus, hit rate calculation in the response verification part was based on only those segments with reduced level of one tone type in which participants reported to perceive either “seg_low” or “seg_high”. For these cases, reporting “seg_high” when the high tones were in fact 25 dB lower in level than the low tones, or vice versa, was counted as a misclassification of the high and low tones. The hit rate thus was defined as the summed time of exclusively pressing the correctly associated button (“seg_high” for reduced level of A, and “seg_low” for reduced level of B) divided by the summed presentation time of segments with one tone type lowered in level in which either “seg_high” or “seg_low” was exclusively pressed. Participants’ data were excluded from all analyses when they did not reach 65% on the thus calculated hit rate; this pertained to 1 out of 20 participants.

#### Replay performance

Hit rate in the replay condition was defined as the summed time of exclusively pressing the correctly associated button divided by the summed time of all replay segments in which any button was exclusively pressed (four buttons available, one correct, three incorrect). Note that the number of percepts that had to be identified in a replay block depended on the reported percepts in the preceding block of the multistable condition; in some cases, certain replay segment types were not played at all. Hit rates are thus calculated by aggregating all segments irrespective of their identity. Participants’ data were to be discarded when they did not reach a hit rate of 65%; no participant was affected by this in Study 1.

#### Button-press characteristics

All analyses were based on *exclusive* button presses, which means that no further button was pressed at the same time. The last response before the block end (in the replay and random conditions) or before the start of the response verification part (in the multistable condition) was discarded because it was interrupted by a physical change or cessation of the tone sequence. The response verification part of the multistable condition was not included in any of the analyses described below.

The *absolute duration* of each response type (i.e., percept in the multistable condition, identified segment in the replay condition, chosen button in the random condition) was calculated by summing up all exclusive press durations of the corresponding button. The *percentage* of each response type was then calculated by dividing its absolute duration by the sum of the absolute durations of all four response types.

The *median dominance duration* per condition was calculated by taking the median of all exclusive button-press durations (aggregating all response types). The median durations were then averaged across participants. A repeated-measures ANOVA with the factor Condition (3 levels: multistable, replay, random) was conducted to compare the median dominance durations across conditions. Follow-up pair-wise comparisons between conditions were conducted via two-tailed, paired *t*-tests. Uncorrected *p* values are reported, and the Bonferroni-corrected alpha level is given.

To quantify randomness of the button-press patterns, the *coefficient of variation (CV)* of all exclusive button-press durations was calculated separately for each condition. CV is defined as the durations’ standard deviation σ divided by the durations’ mean μ:

$$CV=\frac{\sigma }{\mu }$$

A repeated-measures ANOVA with the factor Condition (3 levels: multistable, replay, random) was conducted to compare the CV across conditions. Follow-up pair-wise comparisons were conducted via two-tailed, paired *t*-tests with Bonferroni correction.

#### Pupil data aggregation

The eye-tracking data provided us with 1 sample of pupil diameter (in arbitrary units) per millisecond. The eye-tracker’s built-in software detected blinks and saccades (threshold settings: 35°/s for velocity, 9500°/s^2^ for acceleration). These periods were marked as missing data in the trace of pupil diameters. We further discarded any pupil data obtained at gaze positions outside a central square with an edge length of 10° viewing angle. Moreover, we discarded the first 3 s after each block onset to remove the effects of sequence onset on the pupil diameter. For comparability reasons, the remaining pupil diameter (PD) data of each block was z-standardized. If μ is the mean and σ is the standard deviation over all included samples (PD(t), pupil diameter in arbitrary units) of the block, then the z-standardized pupil trace, zPD(t), can be calculated along all samples PD(t), as follows:

$$zPD(t)=\frac{PD(t)-\mu }{\sigma }$$

Since there is no *a priori* frequency cut-off for pupil signals, we decided prior to data analysis to apply no further filtering or smoothing. To verify that high-frequency fluctuations did not affect the overall result, we reran the complete analysis pipeline described below with a version of zPD that was filtered by a 601 ms wide boxcar filter (the same filter parameters as used for the event-based analysis in S5 Supplement in S1 File). Except for the obvious effect of curves appearing smoother, the result patterns remained entirely unchanged.

We aligned the trace of z-standardized PD values along all recorded button presses in the multistable and random conditions. In the replay condition, we selected only those button presses that constituted the first press after a physical stimulus change, and that happened maximally 2 s after the change. This ensures that we capture the initial response to a replay segment, as opposed to later responses that might reflect additional processes, such as response corrections after detecting an error, or multistable perception occurring after prolonged exposure to a replay segment.

For each included button press, we used the 2 000 samples before and 2 000 samples after the time point of the button press of the PD trace. If two consecutive button presses were closer to each other than 4 s (4 000 samples), this would result in some data being used twice (at the end of the interval for one switch and at the beginning of the interval for the subsequent switch). To prevent such double use, data were assigned uniquely to the closest button press and treated as missing data for the other. For example, if two button presses were spaced apart 3 s, the first of them would have only 1 500 samples after the press, while the second would have only 1 500 samples before the press. There would be missing data for the first 500 ms and the last 500 ms of the analysis interval. This procedure ensures that while avoiding double use of data, the usable portion of the PD data is maximized around the time point of the button press (see S1 Supplement in S1 File).

The resulting PD traces (4 001 samples around each button press) were averaged along the time dimension separately per participant and condition, ignoring missing data on a sample-by-sample basis. As outlined above, missing data could result from blinks/saccades, gaze position outside the 10° square, beginning-of-block, or from avoidance of double use of data (see S1 Supplement in S1 File); and the available data was additionally limited by the number of button presses issued by the participant in the respective condition. If for any condition there was any time point (out of the 4 001) at which a participant did not have any data (i.e., 100% missing data at this time point), all of this individual’s data was excluded from all analyses. No participant was affected by this exclusion criterion in the multistable and replay conditions of Study 1 (see below for more general issues with the random condition).

Average PD traces were baseline-corrected separately per participant and condition by subtracting the average of the zPD of the first 200 samples (i.e., from -2 000 to -1 801 ms relative to the button press) from all 4 001 samples. These z-normalized and baseline-corrected traces are referred to as PD traces hereafter.

#### Maximal amplitude analysis

To statistically compare the amount of pupil dilation around the button press across conditions, the maximal amplitude of the individual PD traces must be extracted per participant and condition. It is difficult to reliably assess the latency of the maximal PD amplitude, and thus the time point for reading out the maximal PD amplitude (highest z value), from the single-participant traces due to their inherent noise level (especially for cases with low numbers of button presses or high proportions of missing data in some samples). To account for this difficulty, we used an approach from the field of event-related brain potential (ERP) component analysis, which is faced with similar signal-to-noise ratio issues: The jackknife approach has been identified as a suitable statistical resampling method that may show a better estimate of the peak amplitude than static averaging [44]. By measuring the maximal amplitude on N sub-samples of N-1 participants (i.e., building averages by consecutively leaving one participant out), we obtained N maximal amplitudes per condition. Maximal amplitudes were compared across conditions as appropriate by a repeated-measures ANOVA with the factor Condition (3 levels: multistable, replay, random) or via two-tailed, paired *t*-tests for pair-wise comparisons. Both the *F* and the *t* values must be corrected to account for the jackknifing, as shown by Ulrich and Miller [45] with

$$F_{corrected}=\frac{F}{{\left(N-1\right)}^{2}}\;\mathrm{and}\;t_{corrected}=\frac{t}{\left(N-1\right)}$$

#### Sample-wise analysis and adjustment for multiple comparisons

In addition to the analysis of maximal amplitudes, the PD traces were examined on a sample-by-sample basis to characterize their temporal trajectories. This involved the comparison of PD traces (4 001 samples) against zero z-score (mean of the given data-set) for each sampling point (1 ms) with a two-tailed *t*-test. The alpha level correction to compensate for this repeated statistical testing was implemented with the false discovery rate (FDR) procedure introduced by Benjamini and Hochberg [46]. Based on the distribution of *p-*values in the given dataset, this procedure generates a corrected alpha level that lies between the uncorrected level of 0.05 and the Bonferroni adjustment, which would result in an overly conservative alpha level of 0.05 / 4 001. Throughout all statistical analyses, the expected FDR is set to 0.05. Significance is denoted whenever the *p*-value remains under the FDR-corrected alpha level, which is reported for each analysis.

## Study 1: Results

### Performance during response verification and replay

We quantified participants’ ability to distinguish the percepts and to use the correct button-response mapping by their hit rates in the response verification part of the multistable condition and by their hit rates in the replay condition. In the response verification part, one participant showed a hit rate of 54.1%, which was below the threshold of 65%; therefore, the following analysis is limited to the remaining 19 (all hit rates at or above 80%) out of 20 participants’ data. The average hit rate of the remaining 19 participants was high (*M* = 92.4% with a standard deviation of 4.8%). In the replay blocks, the hit-rate average was likewise high (*M* = 90.9% with a standard deviation of 6.6%).

### Button-press characteristics

**Fig 3:**
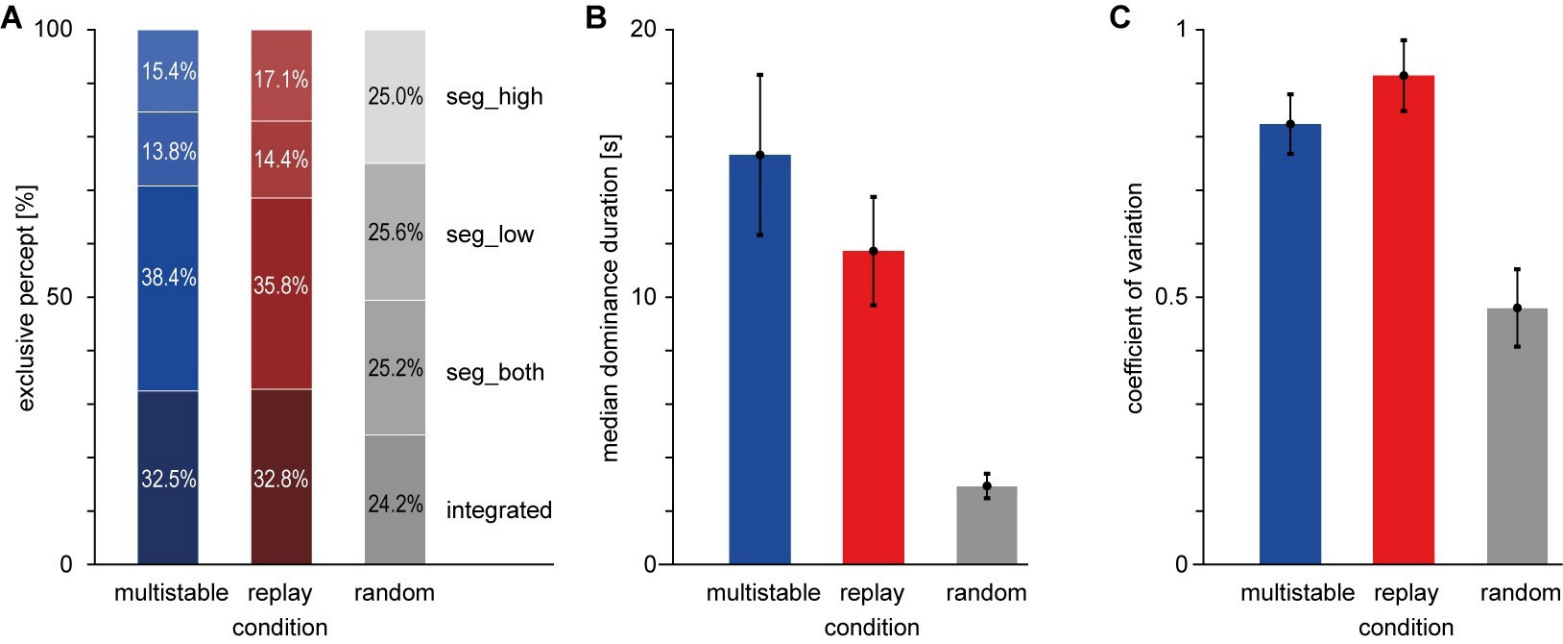
Behavioral data, Study 1. **A**) Percentages of exclusive integrated percept (bottom), exclusive segregated percept with both tones in the foreground (2nd from bottom), exclusive segregated percept with low tone in the foreground (2nd from top), exclusive segregated percept with high tone in the foreground (top). In the random condition, button assignments are arbitrary. **B**) Median dominance duration per participant and condition, mean and standard error of the mean (sem) across listeners. **C**) Coefficient of variation per listener; error bars depict mean and sem across listeners.

Percentages of each response type are shown in Fig 3A. In the multistable condition (ambiguous part only), the reported percentages of each percept were 32.4% for the integrated percept, 38.4% for seg_both, 13.8% for seg_low, and 15.4% for seg_high. In the replay condition, the percentages of identified segments should be similar to those of the multistable condition, which is indeed the case, with 32.8% for the integrated percept, 35.8% for seg_both, 14.4% for seg_low, and 17.1% for seg_high. In the random condition, participants produced approximately equal pressing times for each button, which corresponds to the instruction (24.2%, 25.2%, 25.6%, and 25.0%).

Median dominance durations collapsed across all response types are shown in Fig 3B. They are longest for the multistable condition (*M* = 15.32 s, *SD* = 13.08 s), somewhat shorter for the replay condition (*M* = 11.73 s, *SD* = 8.83 s), and much shorter in the random condition (*M* = 2.94 s, *SD* = 2.00 s). This was confirmed in the ANOVA on median dominance durations, which showed a significant main effect of Condition (*F*(2,36) = 13.4, *p* < .001). Follow-up pair-wise comparisons showed significant differences between the multistable and random conditions (*t*(18) = 3.85, *p* = .001) as well as between the replay and random conditions (*t*(18) = 3.97, *p* < .001), but not (at a Bonferroni-corrected alpha level of 0.05/3) between the multistable and replay conditions (*t*(18) = 2.11, *p* = .049).

The exclusive button-press durations showed a mean coefficient of variation (CV) of 0.82 (*SD* = 0.24) in the multistable, 0.91 (*SD =* 0.29) in the replay and 0.48 (*SD* = 0.31) in the random condition. Randomness of the button-press patterns as quantified by CV was thus notably lower in the random condition, which was confirmed by a significant main effect of Condition in the ANOVA (*F*(2,36) = 42.3, *p* < .001) and by follow-up pair-wise comparisons showing significant differences between the multistable and random conditions (*t*(18) = 7.14, *p* < .001) as well as between the replay and random conditions (*t*(18) = 6.98, *p* < .001). There was also a numerical difference between the CVs in the multistable and replay conditions, which just failed to fall below the Bonferroni-corrected alpha level (*t*(18) = 2.56, *p* = .020).

A CV of 0 implies a perfectly regular pattern, whereas a CV approaching or exceeding 1 corresponds to independence between the timing of subsequent button presses. The low CV in the random condition indicates that participants’ button presses were more regular than instructed, and–unlike intended by design–did not produce a good control for the button-press patterns in the multistable and replay conditions. This undermines comparability of the pupil responses across conditions. In addition to the low variability, an even more problematic aspect was the unexpectedly high rate of button presses in the random condition: despite the instruction to wait for several seconds, the median duration of exclusively holding one button was only 2.94 s. At such fast pace, the pupil response elicited by the button press might not show its full amplitude due to saturation effects, and it also becomes unfeasible to extract an interval of ±2 s around each button press without double use of data and without severe amounts of missing data at the edges (see S1 Supplement in S1 File). For these reasons, we excluded the random condition from the analysis of the pupil responses (for completeness, we refer the reader to the S2 Supplement in S1 File for a descriptive analysis of the pupil traces in the random condition).

### Pupil dilation

Pupil data exclusion based on blinks/saccades, gaze position outside the 10° square, and beginning-of-block led to an amount of missing pupil data of altogether 10.7% (multistable), 12.5% (replay), and 14.7% (random condition). In the PD traces around the button press, the amount of missing data was about equally distributed in the multistable and replay conditions, with a small peak right after the button press (see S1 Supplement in S1 File). This peak results mostly from an abundance of blinks–and to a lesser extent saccades–following the button press, which has previously been observed in visual multistability [47]. In the random condition, the amount of missing data in the 4-s window around the button press was considerably larger towards the beginning and end of the analysis window due to pruning because of too close button presses (S1C Fig in S1 File). Following the criterion that a participant’s data would be excluded from all analyses if an average PD trace in any condition contained a single sample (out of the 4 001) without pupil data, including the random condition in the analysis would have led to the exclusion of three further participants’ datasets. Moreover, average pupil traces of the remaining participants would have been based on very few datapoints towards the edges of the analysis interval. This confirmed our assessment based on the behavioral data that the extraction of robust pupil traces from the random condition would be unfeasible, which led to the aforementioned decision to exclude the random condition from pupil data analysis.

**Fig 4:**
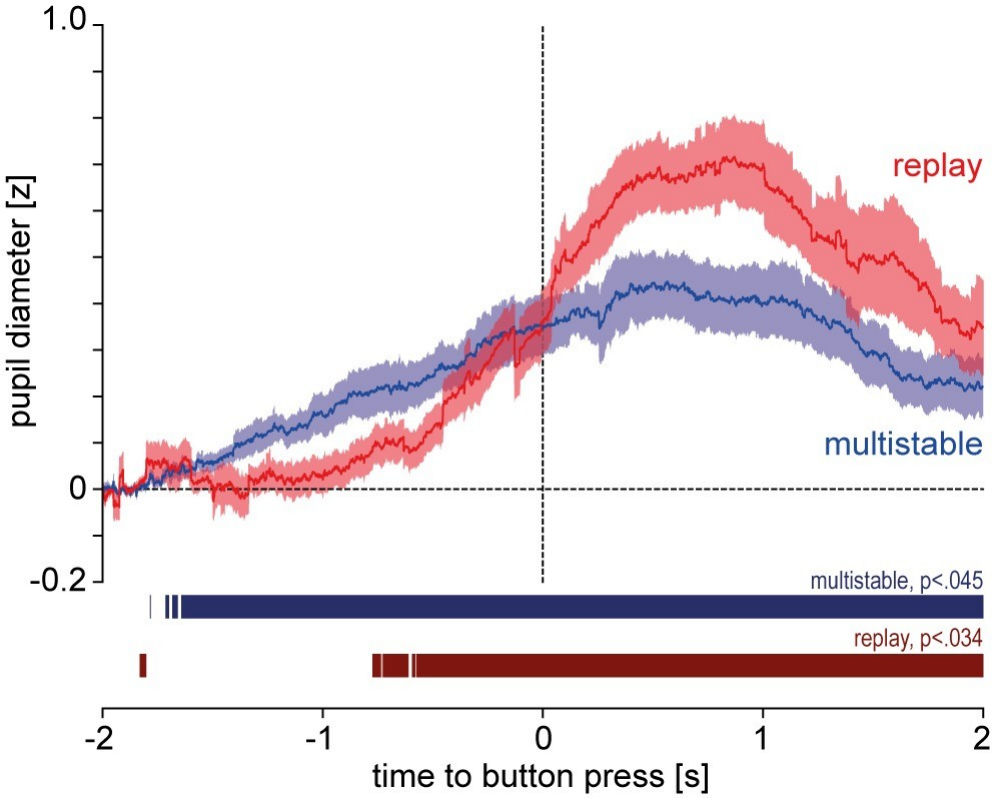
Pupil traces, Study 1. Pupil diameter between 2 s before and 2 s after the button press, z-normalized and subtractively baseline-corrected to 0 mean between [-2 s and -1.8 s]; solid lines: mean, shaded areas: standard error of mean (sem). Colored segments denote periods in which pupil size is significantly different from 0 at an expected FDR of 0.05; adjusted alpha level is denoted on top of the segment.

In the analysis of the pupil data from the multistable and replay conditions, a distinct widening of the PD around the time of the button press was observed in both conditions (Fig 4). This pupil dilation effect was confirmed by a sample-wise comparison of the PD traces against the baseline, zero z-score. In the multistable condition, PD was significantly different from zero continuously at an FDR-corrected alpha level (*p ≤* .045) from 1 642 ms before the button press until the end of the analysis window (2 000 ms after the button press). In the replay condition, PD was significantly different from zero continuously from 574 ms before the button press until the end of the analysis window (*p ≤* .034, FDR-corrected alpha level). A surrogate analysis (see S4 Supplement in S1 File) shows that the pupil dilation effect is abolished when randomly re-positioning the button presses, which rules out statistical artifacts to underlie this finding.

**Fig 5:**
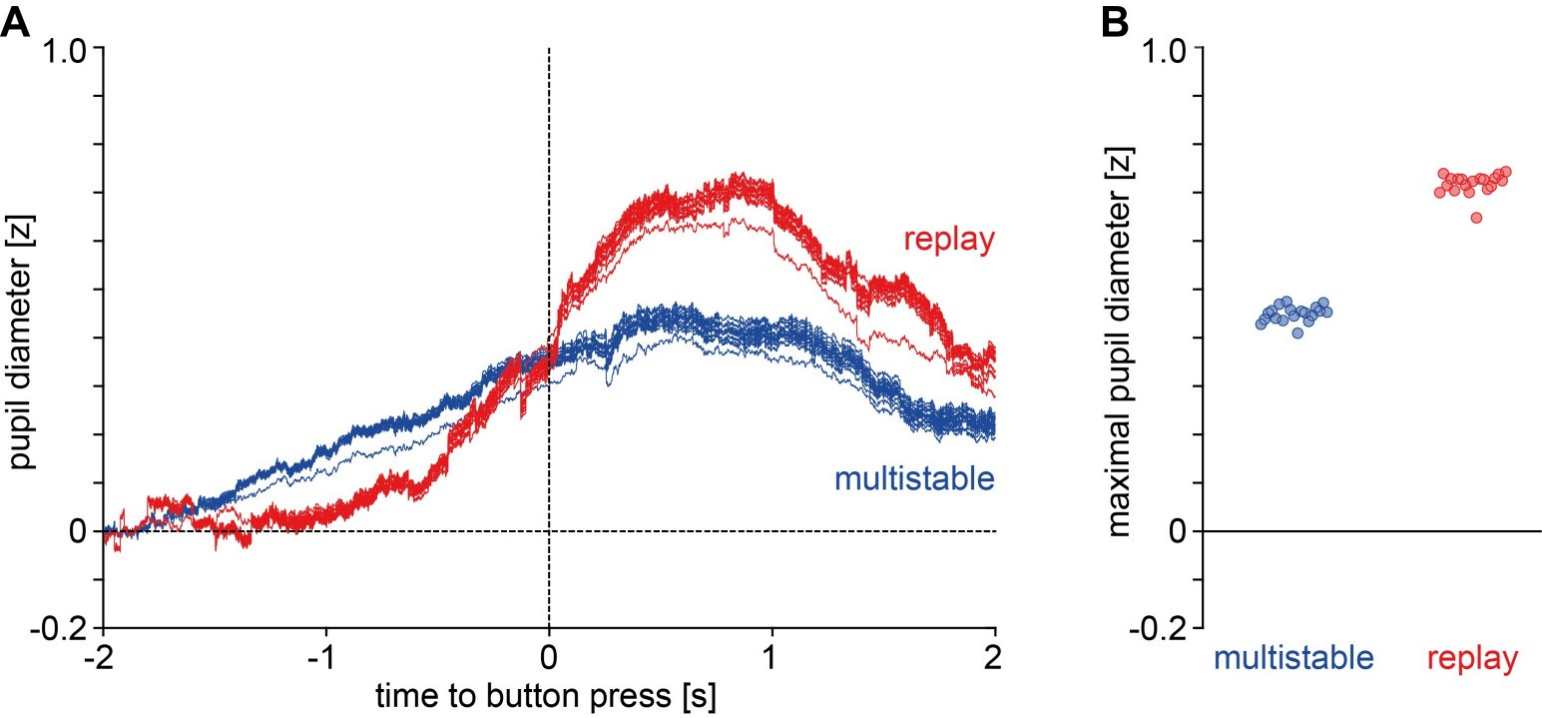
Jackknifing analysis of pupil dilation, Study 1. **A**) Jackknifed traces (19 per condition) and **B**) maximal amplitudes of the jackknifed traces.

The pupil dilation effects in the multistable and replay conditions were compared in terms of their maximal amplitude extracted via jackknifing (Fig 5). Contrary to the hypothesis, maximal amplitude in the replay condition (*M* = 0.71, *SD* = 0.02) was significantly *higher* than in the multistable condition (*M* = 0.45, *SD* = 0.02). This was confirmed by a jackknifing-corrected *t*-test (*t*_*corrected*_(18) = 3.80, *p* < .001).

## Study 1: Discussion

We obtained evidence for a perceptual switch during auditory multistability to be accompanied by a temporary dilation of the pupil. This confirms *hypothesis 1* and corresponds to findings by Einhäuser and colleagues [17] as well as Hupé and colleagues [18] for visual multistability. The pupil dilation commences more than 1.5 s before the button press with which participants indicate their perceptual switch, which is earlier than observed for vision [17, 18]. A plausible explanation is that the time from the start of the perceptual transition to its overt report is typically longer in auditory than in visual multistability [32]. This is because evidence has to be accumulated over some period of time due to the discrete nature of the tone presentation (only 4 tones per second in the present paradigm).

It is important to examine whether the pupil dilation effect indeed relates to the perceptual switch, or whether it is driven by other processes that accompany the report of the perceptual switch. For this purpose, we had included a random control condition [18, 40] and a replay control condition [5, 17]. Unexpectedly, the behavioral result pattern in the random control condition was so different from that in the experimental (multistable) condition that we had to refrain from analyzing the accompanying pupil response because it could not have been interpreted in a meaningful way. This precluded a test of *hypothesis 2*; hence, we cannot infer whether the pupil dilation effect observed during auditory multistable perception is merely due to motor processes (i.e., pressing a report button to indicate the perceptual switch). To be able to test this hypothesis, we developed a slightly adapted approach in Study 2 (see below) that strives for a higher level of comparableness between the button-press behaviors across conditions.

The behavioral measures in the multistable and replay condition were similar by design, since participants reproduced their button-press behavior from multistable perception with the disambiguated replay stimuli. Small numerical differences between these conditions were nevertheless observed in the behavioral measures. A plausible explanation for these differences is that participants needed some time to choose a correct response in the replay condition and that they sometimes noticed a mistake and changed their response during a segment. This shortens the duration of the button presses, and it adds another source of variability and thereby increases the CV. Nevertheless, the effects were modest (not withstanding a Bonferroni-corrected statistical test), and thus the pupil dilation effects can meaningfully be compared to one another.

Contrary to *hypothesis 3*, pupil dilation was larger in response to a perceptual switch caused by a physical change (i.e., in the replay condition) than to an endogenous re-interpretation of physically unchanging input (i.e., in the multistable condition). This result pattern was unexpected, and it is distinct from [18]: In a physical control condition (without the aspect of individual replay), these authors found no significant differences between this control condition and their (visual) multistability condition. Numerically, their effect was larger during multistability than during response to physical stimulus changes, as we had expected to find here. The higher pupil dilation during replay than during multistability observed here might be confounded by two different factors: First, it is possible that the pupil dilation to percept changes in the replay condition is contaminated by the pupil response elicited by the physical stimulus change and thereby enlarged when compared to a percept change during multistability with a physically unchanged stimulus. Indeed, distinct auditory stimulus changes have been shown to have a dilating influence on the pupil [48]. Second, the pupil response in the replay condition might be larger than that in the multistable condition because it is less temporally smeared–that is, the button press might have a tighter (less variable) temporal relation with the actual perceptual change during replay than during multistability. The latter argument would be supported by the observation that the dilation starts more than a second later in the replay than in the multistable condition and then shows a steeper gradient towards the peak.

It is important to note that our analysis of pupil responses is always fixed to the time point of the button press, not to the actual endogenous perceptual switch (multistable condition) or to the exogenously caused perceptual switch (replay condition) or to the decision to randomly press another button (random condition)–because these events are not overtly observable. Therefore, the explanation based on temporal smearing is difficult to test in a straightforward manner. We thus modified our paradigm to include an experimental test of the first explanation: By isolating the effect of the physical stimulus change on the pupil response in Study 2 (see below), we can correct the pupil response during replay for pupillary components evoked by this change.

## Study 2: Introduction

Study 2 reproduced Study 1 with a fresh set of participants and with two advancements in the experimental paradigm, as detailed below. We expected to replicate the pupil dilation in response to a perceptual switch during auditory multistability (*hypothesis 1*) and to characterize this dilation in more detail than Study 1 allowed us to. For testing hypotheses 2 and 3 more rigorously, we introduced two major improvements. First, by careful adaptation of the instructions, we sought to evoke a button-press pattern in the random condition that is more similar to the other conditions to be able to compare the pupil responses in a meaningful way. This would allow us to address *hypothesis 2*, namely that the pupil dilation during auditory multistability exceeds the one associated with a mere button press (random condition).

Second, by introducing an additional control condition, we sought to compensate for a possible contamination of the pupil response by physical stimulus changes during stimulus replay. More specifically, in Study 2, we applied two different replay conditions: replay-active, which is identical to the replay condition of Study 1, and replay-passive, which involves the same stimulus presentation as replay-active but without any overt response required from the participants. The new replay-passive condition allows us to estimate the effect of physical stimulus changes on the pupil response, which can then be subtracted from the pupil responses in the replay-active condition. This leads to a re-examination of *hypothesis 3*: If the unexpected result pattern observed in the comparison of the multistable and replay conditions was due to physical stimulus change effects, pupil dilation in the corrected replay condition should no longer exceed pupil dilation during multistability–if, on the other hand, it was due to temporal smearing, the result should remain unchanged.

## Study 2: Materials and method

All procedures, setup and stimulus details were identical to Study 1 unless otherwise denoted.

### Participants

26 healthy volunteers (age [mean ± standard deviation]: 22.3 ± 3.01 years; 8 male, 18 female; 5 left-handed, 20 right-handed, 1 both-handed) participated in Study 2. Data collection for one additional volunteer was commenced but had to be cancelled for technical reasons.

### Setup

To improve data quality, the eye-tracking camera and IR emitter were re-positioned relative to Study 1.

### Visual stimuli

During each type of auditory stimulation, the display showed a combination of bulls eye and cross hair as recommended by Thaler, Schütz, Goodale, and Gegenfurtner [49] for experiments requiring stable fixation. The fixation target was presented in black in the center of the screen (luminance: 0.1 cd/m^2^; size: 1.2° degrees of visual angle). Participants were asked to direct their gaze at the fixation target throughout.

The screen’s background luminance (on which the fixation cross was presented) was optimized separately for each participant by a procedure at the very beginning of the study. After Eyelink calibration (9 points covering the central area of the display), for a duration of 5 s each, the whole display emitted one of 13 luminance levels (ascending order): 0.1, 12.5, 25, 37.5, 50, 62.5, 75, 87.5, 100, 112.5, 125, 137.5, 304.0 cd/m^2^, whereby the participant fixated the fixation target in the middle of the screen. For each luminance level, the last second of the recorded pupil diameter was examined, and a luminance level was picked that was close to the midpoint of the pupil diameters evoked by the lowest and highest luminance presented. The aim of this procedure was to choose a luminance level for the experiment that would give an intermediate pupil diameter to prevent ceiling and floor effects in the pupil response.

### Procedure

The experiment was divided into nine blocks. Depending on the parity (even or odd) of the participant number, participants passed the experiment along one of two orders (I or II). Both orders contained the same number of blocks per type, but in a different succession to prevent order effects. The first (1) and last (9) blocks belonged to the random condition, and blocks 2, 5 and 8 were always of the multistable condition. The positioning of the blocks of the replay-passive and replay-active conditions differed between orders I and II. In order I, blocks 3 and 7 were replay-active blocks (and thus blocks 4 and 6 were replay-passive blocks); complementary to that, in order II, blocks 4 and 6 were replay-active blocks (and thus blocks 3 and 7 were replay-passive blocks). As in Study 1, training blocks were situated before the first appearance of the multistable condition (block 2) and the replay-active condition (block 3 or 4). The four different button mappings from Study 1 were systematically crossed with the alternating block orders (I and II) to counterbalance all unique combinations.

#### Random condition

The change of the random condition relative to Study 1 pertained to the instructions given to participants and to a check of their fulfillment in the first block of the experiment. With the purpose of yielding median button-press durations longer than in Study 1 (and long enough to enable an analysis window of 4 s width), the instructions were shortened to focus the attention on the most important aspects, namely that participants should always hold only one button, and that they should hold it for several seconds before switching to another button. It was no longer mentioned that they should refrain from any planned order in the button sequence, nor from any rhythm in the timing of presses and releases, because these various requirements might have detracted them from the simple instruction not to change the buttons too quickly. The envisaged button-press duration of more than 4 s was additionally enforced by repeating the first random block once if the median button-press duration was below 4 s on the first attempt. In these cases, only the repeated block was used for further analysis. The last block of the experiment (i.e., the second block of the random condition) was not repeated, even if the criterion (median < 4 s) was violated.

#### Replay-active condition

The replay-active condition in Study 2 corresponded to the replay condition in Study 1 in all respects.

#### Replay-passive condition

The replay-passive condition was constructed in the same way as the replay-active condition, with independent random shuffling for the replay-passive and -active blocks that were based on the same block of the multistable condition. The core difference between replay-passive and -active pertained to task and instructions. In the replay-passive condition, participants were not asked to report the current segment. Instead, they were instructed to focus their attention on the auditory sequence and to maintain fixation. The physically different segments (int, seg_both, seg_low, seg_high) that would be played were introduced just as in the replay-active condition. The button box was removed from the participants’ access, and no further training (as for the multistable and replay-active condition) was applied.

### Data analysis

The data analysis was identical to Study 1, with an extension for the corrected replay condition.

#### Correction of the replay condition for effects of stimulus change

To obtain a pupil trace for the replay condition that factors out the effect of stimulus change, we computed *replay-corrected* traces by a point-wise subtraction of data of the replay-passive condition from the replay-active condition as follows: The PD traces in the replay-passive condition were aligned to the onset of the physical stimulus change. Segments from -2 s to +4 s relative to the stimulus change were extracted (ignoring missing data on a sample-by-sample basis as in the other conditions, see Study 1 for details), and were averaged within each participant to yield this participant’s average pupil response to a change in the auditory stimulus without an overt response. This single-subject average PD trace of 6 001 samples length was baseline-corrected by subtracting the average of the first 200 samples (i.e., from -2 000 to -1 801 ms relative to the stimulus change) from all 6 001 samples.

The pupil response in the replay-active condition was extracted time-locked to the button press for each button press that met the criteria laid out for the replay condition in Study 1. Then the baseline-corrected average replay-passive trace was aligned with the stimulus change preceding this button press. (This stimulus change falls–by the defined criteria–within the 2 s prior to the button press, thus the [-2 s to 4 s] interval of the replay-passive condition suffices for the correction.) The thus corrected traces were averaged and baseline-corrected as in the replay condition of Study 1. They are referred to as replay-corrected traces throughout. The replay-corrected traces were then analyzed analogously to the replay condition of Study 1 in terms of deviation from the baseline and in terms of comparison of the maximal amplitude (via jackknifing) between all three conditions (multistable, replay-corrected, and random). A comparison between the uncorrected replay-active traces and replay-corrected traces are given in S3 Supplement in S1 File.

## Study 2: Results

### Data exclusion

Out of the 26 participants, 7 were excluded from further analysis because they met one or more exclusion criteria: one participant showed a hit rate of 21.6% (well below the threshold of 65%) in the response verification part of the multistable condition, four participants showed hit rates below 65% in the replay condition (46.4%, 52.4%, 60.0%, 64.4%). For five participants, PD traces could not be averaged in at least one of the conditions because there was at least one single sample with zero PD traces contributing. The exclusion criteria were partly overlapping, leading to a loss of 7 datasets altogether.

### Performance during response verification and replay

The remaining 19 participants showed a high hit rate of 90.4 ± 5.5% in the response verification part of the multistable condition and a likewise high hit rate of 91.3 ± 6.1% in the replay-active condition.

### Button-press characteristics

**Fig 6:**
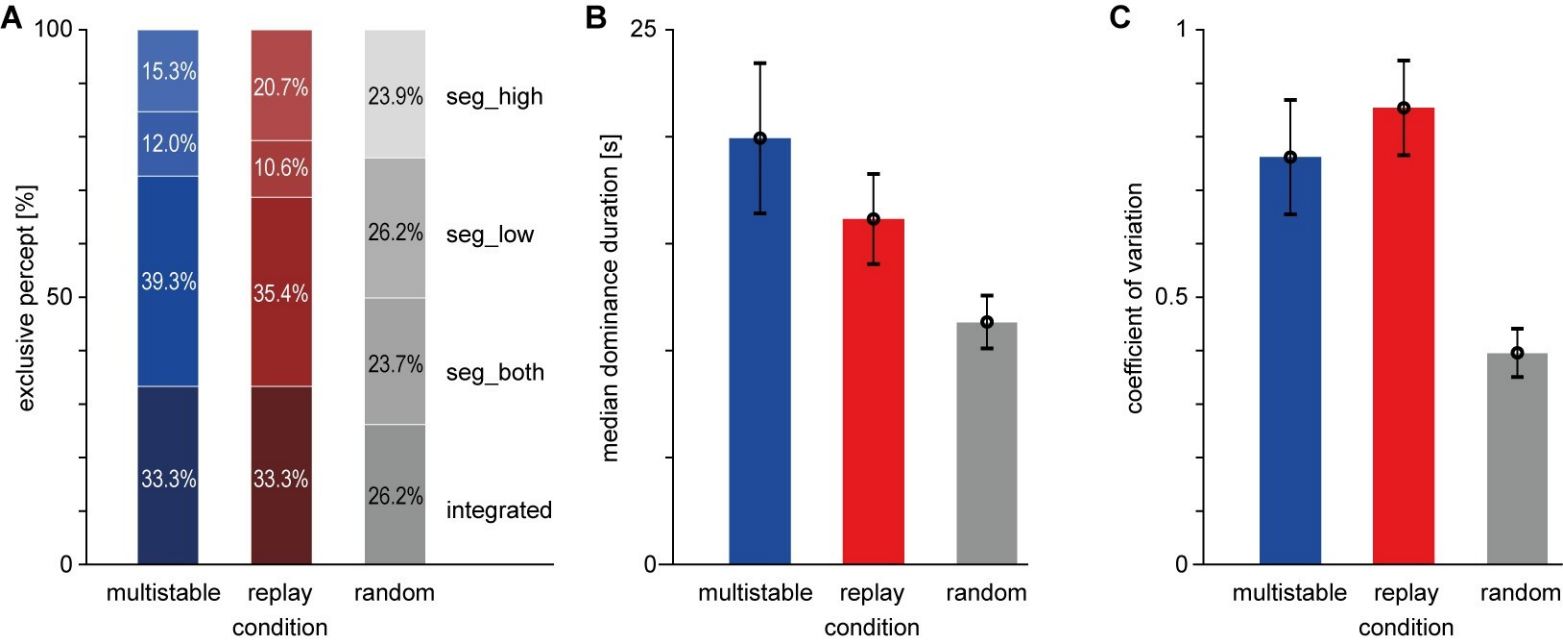
Behavioral data, Study 2. **A**) Percentages of exclusive integrated percept (bottom), exclusive segregated percept with both tones in the foreground (2nd from bottom), exclusive segregated percept with low tone in the foreground (2nd from top), exclusive segregated percept with high tone in the foreground (top). In the random condition, button assignments are arbitrary. **B**) Median dominance duration per participant and condition, mean and standard error of the mean (sem) across listeners. **C**) Coefficient of variation per listener; error bars depict mean and sem across listeners.

Percentages of each response type are shown in Fig 6A. In the multistable condition, the reported percentages of each percept were 33.3% for the integrated percept, 39.3% for seg_both, 12.0% for seg_low, and 15.3% for seg_high. In the replay condition, the percentages of identified segments should be similar to those of the multistable condition, which is the case as in Study 1, with 33.3% for the integrated, 35.4% for seg_both, 10.6% for seg_low, and 20.7% for seg_high. In the random condition, participants again produced approximately equal pressing times for each button (26.2%, 23.7%, 26.2% and 23.9%).

Median dominance durations collapsed across all response types are shown in Fig 6B. As in Study 1, median dominance durations were slightly longer in the multistable condition (*M* = 19.93 s, *SD* = 15.30 s) than in the replay condition (*M* = 16.16 s, *SD* = 9.20 s). They were again shorter in the random condition (*M* = 11.34 s, *SD* = 5.37 s), but the difference was not as extreme as in Study 1, where median dominance durations in the random condition were almost four times lower (*M* = 2.94 s) than in Study 2. Nevertheless, a significant difference between conditions was confirmed in the ANOVA on median dominance durations observed in Study 2 (*F*(2,36) = 5.54, *p* = .008). Follow-up pair-wise comparisons showed differences between the multistable and random conditions (*t*(18) = 2.60, *p* = .018) as well as between the replay and random conditions (*t*(18) = 2.39, *p* = .028), both of which fall slightly above the Bonferroni-corrected alpha level. No significant difference between the median dominance durations in the multistable and replay conditions was observed (*t*(18) = 1.67, *p* = .112).

The exclusive button-press durations showed a mean coefficient of variation (CV) of 0.76 (*SD* = 0.47) in the multistable, 0.85 (*SD =* 0.39) in the replay and 0.40 (*SD* = 0.20) in the random condition. Randomness of the button-press patterns as quantified by CV was thus again notably lower in the random condition, which was confirmed by a significant main effect of Condition in the ANOVA (*F*(2,36) = 14.12, *p* < .001) and by follow-up pair-wise comparisons showing significant differences between the multistable and random conditions (*t*(18) = 3.45, *p* = .003) as well as between the replay and random conditions (*t*(18) = 5.48, *p* < .001), but not between the multistable and replay conditions (*t*(18) = 1.12, *p* = .276). It should be noted that the low CV in the random condition of Study 2 does not indicate a violation of instructions because–unlike for Study 1 –the instructions did not ask participants to refrain from any rhythm in the timing of presses and releases. Importantly, the median interval between any two consecutive button presses in the random condition was now in a range that made the analysis of pupil effects feasible.

### Pupil dilation

Pupil data exclusion based on blinks/saccades, gaze position outside the 10° square, and beginning-of-block led to an amount of missing pupil data of altogether 7.6% (multistable condition), 8.1% (replay-active), 7.6% (replay-passive), and 9.2% (random condition). In the PD traces around the button press or stimulus change, the amount of missing data was about equally distributed, with a small peak right after the button press in the three conditions with response requirement (see S1 Supplement in S1 File).

**Fig 7:**
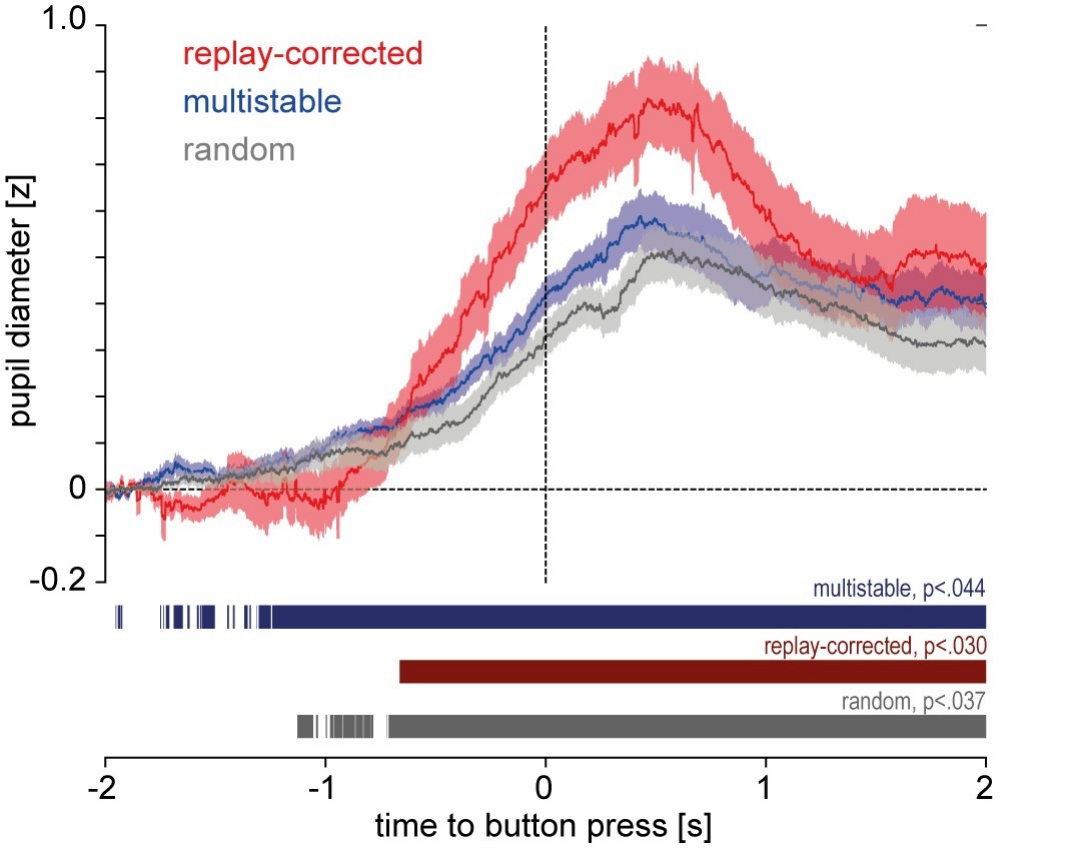
Pupil traces, Study 2. Pupil diameter between 2 s before and 2 s after the button press, z-normalized and subtractively baseline-corrected to 0 mean between [-2 s and -1.8 s]; solid lines: mean, shaded areas: standard error of mean (sem). Colored segments denote periods in which pupil size is significantly different from 0 at an expected FDR of 0.05; adjusted alpha level is denoted on top of the segment.

Fig 7 shows a distinct widening of the PD around the time of the button press in all three conditions (multistable, replay-corrected, and random). The pupil dilation effect was confirmed in all three conditions by a sample-wise comparison of the PD traces against the baseline, zero z-score. In the multistable condition, PD was significantly different from zero (*p ≤* .043, FDR-corrected alpha level) continuously from 1 240 ms before the button press until 2 000 ms after it, corresponding to the end of the analysis window. In the replay-corrected condition, PD was significantly different from zero continuously from 659 ms before the button press until 2 000 ms after it (*p ≤* .030, FDR-corrected alpha level). In the random condition, PD was significantly different from zero continuously from 691 ms before the button press until 2 000 ms after it (*p ≤* .037, FDR-corrected alpha level). A surrogate analysis (see S4 Supplement in S1 File) shows that pupil dilation is abolished when randomly re-positioning the button presses, which rules out that statistical artifacts underlie the effect.

**Fig 8:**
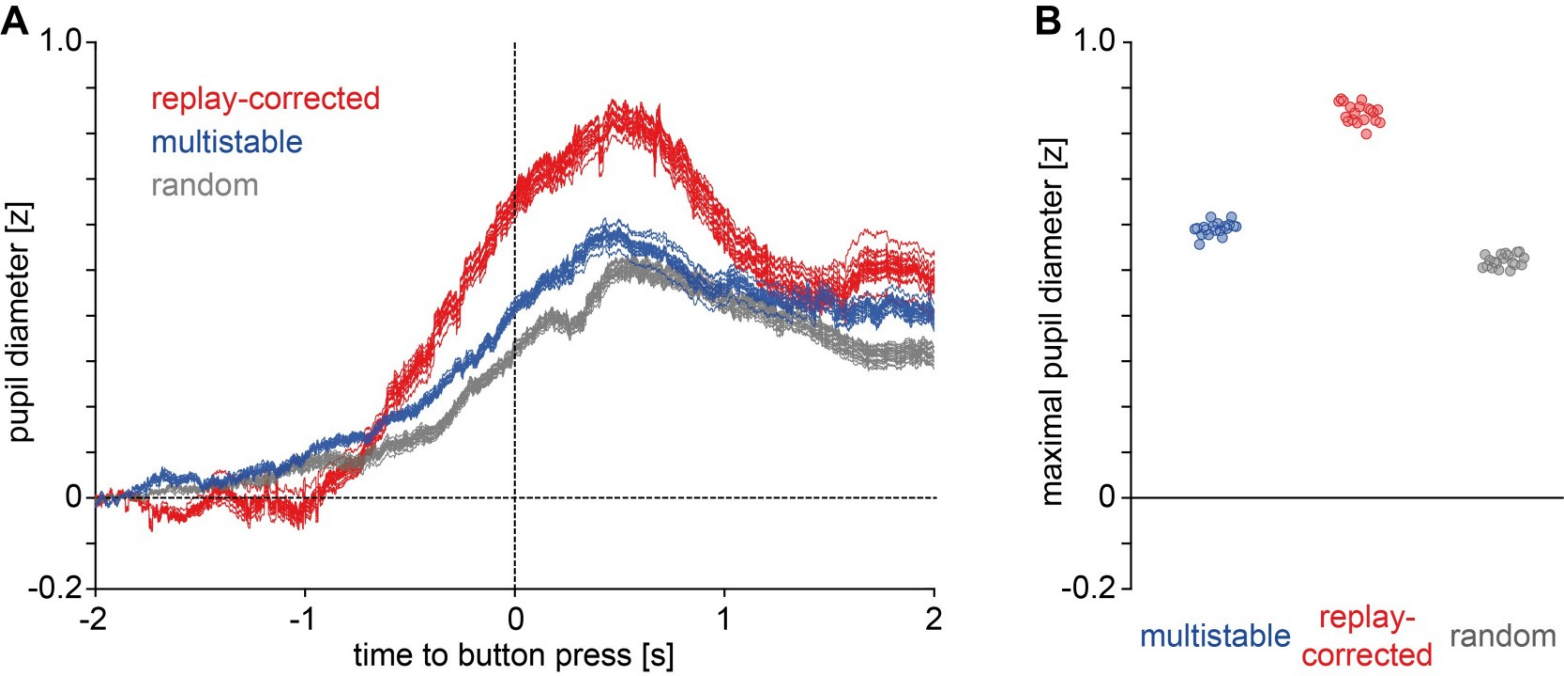
Jackknifing analysis of pupil dilation, Study 2. **A**) Jackknifed traces (19 per condition) and **B**) maximal amplitudes of the jackknifed traces.

The pupil dilation effects in the multistable, replay-corrected and random conditions were compared in terms of their maximal amplitude extracted via jackknifing (see Fig 8). Maximal amplitudes were 0.59 (*SD* = 0.01) in the multistable, 0.84 (*SD* = 0.02) in the replay-corrected and 0.52 (*SD =* 0.01) in the random condition. The jackknifing-corrected ANOVA confirms a significant effect of Condition on the maximal amplitudes (*F*_*corrected*_(2,36) = 7.06, *p* = .003). Follow-up pair-wise comparisons with jackknifing-corrected *t*-tests showed a significant difference between the replay-corrected and random conditions (*t*_*corrected*_(18) = 3.32, *p* = .004), a difference that fails to meet the Bonferroni-corrected alpha level between the multistable and replay-corrected conditions (*t*_*corrected*_(18) = 2.44, *p* = .025), and no significant difference between the multistable and random conditions (*t*_*corrected*_(18) = 1.08, *p* = .297).

## Discussion

As in Study 1, we found evidence for a perceptual switch during auditory multistability to be accompanied by a temporary dilation of the pupil. This confirms *hypothesis 1* and is in line with pupil dilation during visual multistability [17, 18]. Again, the pupil dilation commences more than a second before the button press with which participants indicate their perceptual switch, which might be due to prolonged evidence accumulation during auditory (as opposed to visual) perceptual decisions for such stimuli.

In Study 2, we can relate the pupil dilation effect during auditory multistability to other processes that accompany the report of the perceptual switch. Specifically, pupil data from the random control condition [18] could be meaningfully analyzed in Study 2 thanks to the longer durations of participants’ button presses (median 11.34 s as opposed to 2.94 s in Study 1). Analysis of the pupil data during random button pressing shows that reliable pupil dilation around the button press is elicited in this condition as well, and that the maximal amplitude of the pupil dilation does *not* differ from that observed during perceptual multistability. This disconfirms *hypothesis 2* and is distinct from results of a similar comparison for visual multistability [18], where the amplitude of pupil dilation during the motor-control condition amounted to only 70% of the amplitude during multistability (i.e., there was a genuine component of the perceptual switch). In the worst case, the present result indicates that pupil dilation during auditory multistability is simply an artifact of pressing a response button. Alternatively, our random control condition might have been too demanding for participants, who had to choose between four possible response buttons (obviously making efforts to press each of them for the same amount of time) while keeping the timing requirements and trying to produce random behavior. Such task demands would increase the pupil response at the time of the decision [50]. Similarly, the decision as such may elicit a pupil dilation [27, 28]. In either case, the random condition would not be suitable for isolating the mere motor act but would come with its own deliberation and decision processes.

Random button-press control conditions are not yet routinely applied in experiments involving auditory multistable perception (see [40] for an exception). In the present studies, we made an effort to keep the random (control) condition as similar as possible to the multistable (experimental) condition. Future studies should systematically compare different instructions for random button pressing with the aim of producing similar behavioral outcome while avoiding the cost of involving too many cognitive processes on top of pressing a button. Another way to separate button-press effects from perception and decision processes would be to use a no-report paradigm that allows for continuous monitoring of participants’ momentary percept without the need of behavioral reporting [14]. Yet since no-report paradigms for visual [51, 52] and auditory [32] multistability often rest on specific patterns (such as the optokinetic nystagmus, OKN) obtained via eye-tracking, this might be challenging to reconcile with undistorted pupil dilation measurements. Nonetheless, a recent study [53] used such a no-report paradigm in binocular rivalry and found the pupil dilation at a perceptual switch to be mostly task-related, while the perceptual transition itself was characterized by a constriction prior to reporting the switch. Importantly, in the present data, the significant difference in pupil dilation between the random and replay conditions clearly indicates that pupil dilation carries a genuine perceptual component for auditory stimuli.

Behavioral measures in the replay and multistable conditions were statistically indistinguishable in Study 2. The comparison of the pupil data from these two conditions thus remains valid. Similar to Study 1, and again contrary to *hypothesis 3*, pupil dilation was larger to a perceptual switch caused by a physical change (i.e., during replay) than to an endogenous re-interpretation of physically unchanging input (i.e., during multistability). In Study 2, we corrected the pupil data of the replay condition by the pupil dilation caused by the physical stimulus change (through the passive-replay condition). Indeed, physical stimulus changes did produce a non-negligible confound on the pupil dilation amplitude (see S3 Supplement in S1 File), which we eliminate by our subtraction approach. The resulting replay-corrected trace reflects cognitive components that are shared with those reflected in the pupil trace from the multistable condition: namely, making a decision (i.e., classifying the new percept and identifying the button that is associated with it) and pressing a button. Since the multistable condition carries the additional component of an endogenous perceptual switch, we had expected to find higher pupil dilation amplitudes here than during replay (such data pattern was observed numerically, though without a significant difference, in [18] for visual multistability). Since we can rule out possible confounds by physical stimulus changes in Study 2, we remain with the possibility that the pupil response in the replay condition is larger than that in the multistable condition because it is less temporally smeared. In other words, the interval from the perceptual change to the execution of the button press might be less variable during replay than during multistability because the perceptual transition is more distinct. As in Study 1, the steeper gradient of the pupil dilation during replay than during multistability supports this possibility.

Future studies should explicitly address effects of temporal smearing and their possible influence on the pupil response during auditory multistability and stimulus replay. As a first analysis with our present data, we defined pupil dilation “events” [54, 55] in each time series (S5 Supplement in S1 File). We found individual pupil-dilation events in the second prior to the button press to be associated with more rapid dilation (i.e., the pupil size having a larger slope) in replay than in the other conditions. This suggests that temporal smearing is not the only source of the difference in the average trace. However, for Study 2, we also observed more switches to be accompanied by pupil-dilation events in replay than in the other conditions (S5C Fig in S1 File). This leaves the possibility that some button presses were not well locked to an actual perceptual change (i.e., the perceptual change happened before the 1-s interval preceding the button press used for this analysis), which would result in an extreme variant of temporal smearing. One possible experimental approach to address this could involve comparing listeners with different degrees of training in reporting their auditory multistable perception, based on the idea that temporal smearing diminishes with training [32, 34]. With the amount of training, the button press should be better synchronized to the actual perceptual change. If the temporal smearing account is valid, the average pupil dilation amplitude during multistability should thus increase with training, as should the fraction of pupil-dilation events immediately preceding the button-press. A lack of experience with the stimulus that yields imprecise synchronization between button press and perceptual change could also explain why more robust pupil dilation has been reported for visual multistability. The respective studies [17, 18] used types of visual multistability that typically feature a sharp perceptual transition between distinct interpretations even in untrained observers. It is conceivable that results on visual paradigms with more gradual transitions, most notably binocular rivalry [56–58], will yield results qualitatively more similar to our auditory results. Indeed, a recent study found the pupil dilation after a switch in binocular rivalry to be similar to the dilation in a closely matched replay condition [53]. Another possibility to assess the effects of temporal smearing is to introduce gradual rather than abrupt changes during the replay condition to mimic the gradual nature of perceptual transitions during multistability. Here, the pupil dilation amplitude observed during replay should decrease. In both cases, if *hypothesis 3* holds, the amplitude difference in the pupil dilation between multistability and replay should vanish and eventually reverse–which would indicate a genuine contribution of the perceptual switch to pupil dilation.

The replay and random conditions were both designed as control conditions for the multistable condition, and the focus of the study was not on comparing the control conditions with one another. Nonetheless, the larger pupil dilation in the replay-corrected than random condition warrants discussion. The motor requirements in both conditions were identical, thus the difference must stem from the “decision” preceding the button press. In the random condition, this decision was relatively unconfined (though participants possibly try to comply with assumed requirements, see above). In the replay condition, the decision involved perceptual components (classifying the new percept) and percept-response mapping (identifying the correct button), and participants knew that their button press will be judged as correct or wrong. Even though the effects of the physical stimulus change are subtracted out of the replay-corrected trace, the remaining components (percept classification, percept-response mapping, expectation of an external evaluation) are not mimicked in the replay-passive condition, and thus they remain different between the replay-corrected and random traces. It is conceivable that the elevated perceptual and evaluative components lead to larger pupil dilation in the replay-corrected than in the random traces. In addition, similar to the difference between replay and multistable condition, the volitional decision in the random case might be less discrete than the response to a stimulus change, resulting in the pupil response in the random condition to be more smeared out and therefore having a smaller maximum on average than in the replay-corrected condition (see above for discussion of temporal smearing).

When relating the pupillometry effects observed here to other psychophysiological measures associated with perceptual multistability, it is important to distinguish between studies that identify physiological correlates of specific percepts (for example with fMRI in visual multistability [59]) from studies that address correlates of the switching process itself. In auditory multistability, correlates of switching have been studied with EEG by Higgins and colleagues [60] and with fMRI by Kashino and Kondo [61]. In visual multistability, switching has been examined with fMRI [14–16, 62], MEG [63] and EEG [64, 65]. All these studies have in common that they specifically look for physiological correlates that are modality-specific. Pupillometry, in contrast, has the potential to tap into mechanisms that are shared across modalities and–due to their physiological roots in the brainstem [66]–hard to access with other methods.

As a side-note, the stimulus used for the replay conditions in Study 1 and 2 proved to be easily identifiable after a short amount of training for most participants (42 out of 46 across both studies). The disambiguation via chirp sounds might be an interesting option for other studies of auditory multistable perception using variants of the auditory streaming paradigm [30]. Such studies are often in need of response validity checks or catch trials [40]. Catch trials are mostly implemented by extremely small or large frequency differences between the ‘A’ and ‘B’ sounds to promote integrated or segregated percepts, respectively. Yet such catch trials may not be advisable if the main experimental manipulation also includes a variation of ‘A’-‘B’ frequency difference because they might be too strongly suggestive of seemingly ‘correct’ behavioral responses during multistability. The disambiguation used in the present studies has the advantage of leaving the frequency difference of the core part of the sound intact, which might be a useful alternative for future studies.

## Conclusions

Taken together, we conducted two studies that demonstrate a transient pupil dilation accompanying perceptual switches during auditory multistability. This complements previous observations for visual multistability [17, 18]. Unlike for visual multistability [18], it is not yet possible to decompose the pupil dilation effect during auditory multistability into different components reflecting perceptual versus motor processes. Based on our findings, we developed specific recommendations for future studies that would allow such decomposition by using improved control conditions. Isolating an unequivocal perceptual component of pupil dilation during auditory multistability would be important to interpret the pupil as a marker of shared mechanisms contributing to perceptual multistability across modalities [17].

## Supporting information

S1 File (PDF)
