# Supplementary material for: Pupillometry in auditory multistability
Source: PLoS One. 2021 Jun 4;16(6):e0252370. doi: 10.1371/journal.pone.0252370 (PMC8177413; doi:10.1371/journal.pone.0252370)
Supplement: S1 File — (PDF) [file pone.0252370.s001.pdf]

Supplement for "Pupillometry in auditory multistability"

Jan Grenzebach, Thomas G.G. Wegner, Wolfgang Einhäuser, Alexandra Bendixen

Supplement S1: Missing data

The core analysis of the two studies reported here rests on the examination of pupil diameter (PD) changes in a window from 2 s before to 2 s after each button press. As outlined in the main text, several factors can lead to missing data around a button press. These include blinks/saccades, gaze position outside the 10° square, beginning-of-block, and pruning to avoid double use of data. To assess the robustness of the data underlying the average PD traces, the time course of the amount of missing data per condition was examined (Fig S1).

Although the amount of missing data is substantial (~20%), this is typical of such paradigms with high task demands and fixation instruction, and the consistency of the sample-wise statistical testing indicates that results were not systematically distorted. There is a small peak of missing data right after the button press in most conditions, which mainly results from an increase in blinks after the button press and – to a lesser extent – from an increase in saccades. This is similar to the pattern that has previously been described for visual multistability [1]. The only condition with a severe and unexpected amount of missing PD data was the random condition of Study 1. The massive data loss towards the edges of the analysis window was due to pruning because of too close button presses (median 2.9 s button-press duration relative to a 4 s analysis window). Consequently, the PD data from this condition were excluded from further analysis.

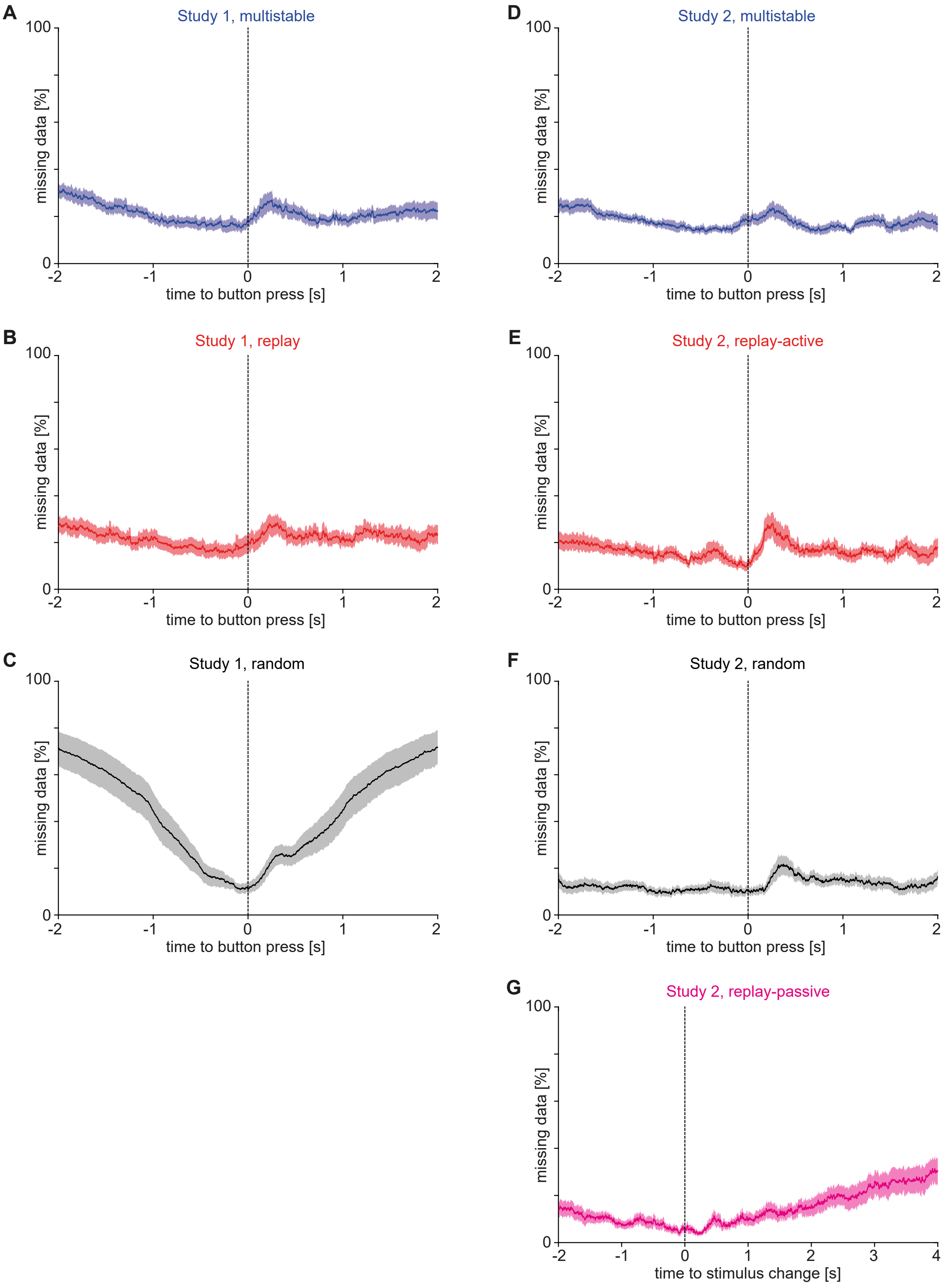

Fig S1. Missing data. Amount of missing pupil diameter data between 2 s before and 2 s after the button press (panels A-F) or stimulus change (panel G), separately for each condition in Study 1 (left column) and Study 2 (right column). Missing data refers to the percentage of individual pupil traces that had to be excluded from the pupil diameter analysis relative to the number of epochs that contributed at least one data point.

Supplement S2: Pupil traces in the random condition of Study 1

To verify that the exclusion of Study 1's random condition does not distort the overall results, the PD trace in this condition was analyzed and compared with the multistable and replay conditions on a descriptive level (Fig S2). It is important to note that only from -1.246 s to +1.246 s relative to the button press, PD data are available for all 19 participants. The severe loss of data towards the edges of the analysis window in the random condition (see Fig S1) leads to discontinuities in the PD trace that must not be interpreted as representing actual "jumps" in pupil size. Rather, it is due to individual participants not contributing anymore to the group-average PD trace, as well as to severe reductions in the number of epochs in those participants who do still contribute to the group average. The unavailability of PD data for some participants in the interval used for baseline correction (-2 s to -1.8 s) made a baseline correction unfeasible for the group-average PD trace of the random condition.

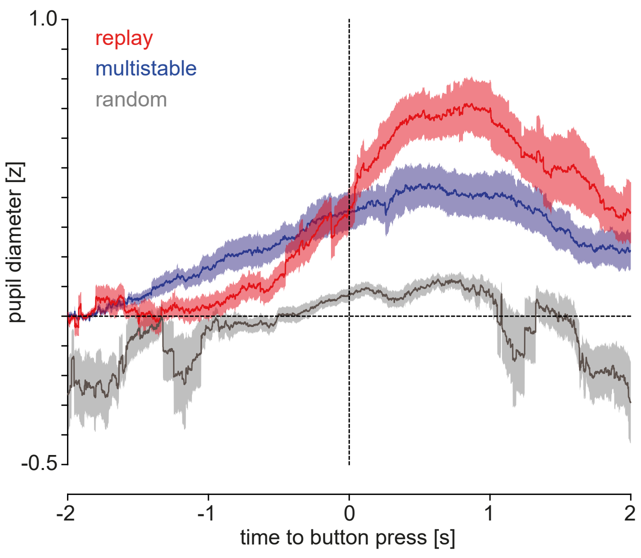

Fig S2. Pupil traces in the random condition of Study 1. Pupil diameter data in the random condition of Study 1 between 2 s before and 2 s after the button press (gray). Pupil diameter in the replay (red) and multistable (blue) conditions are plotted for comparison. Notation as in Fig 4 of the main text. For the replay and multistable conditions, every participant contributes PD data to each time point. For the random condition, some single-participant PD traces are discontinuous towards the edges of the

analysis window, leading to distortions of the group-average PD trace and rendering baseline correction unfeasible in this condition.

At a descriptive level, the pupil response in the random condition is shallower than in the multistable and replay conditions. This appears to be in line with *hypothesis 2*, but should not be interpreted as evidence with respect to this hypothesis. In addition to the above-mentioned issues with PD data availability, the fast pace of the button presses might lead to saturation of the pupil response, as it leaves insufficient time for recovery to baseline. This, in turn, might result in a smaller apparent response of the pupil to any given button press and thus confound the analysis. Hence, we refrain from inferences about the pupil response in the random condition of Study 1. Based on these observations, we modified the instruction for and the design of the random condition of Study 2.

Supplement S3: Correction of the replay condition in Study 2

To separate effects of physical stimulus changes from effects of perceiving and responding to a stimulus change in the replay-active condition of Study 2, a replay-passive condition without response requirements was added as a control. Pupil data from the replay-passive condition were point-wise subtracted from the pupil data of the replay-active condition to yield the replay-corrected pupil trajectory (Fig S3A, see main text for details of the subtraction procedure).

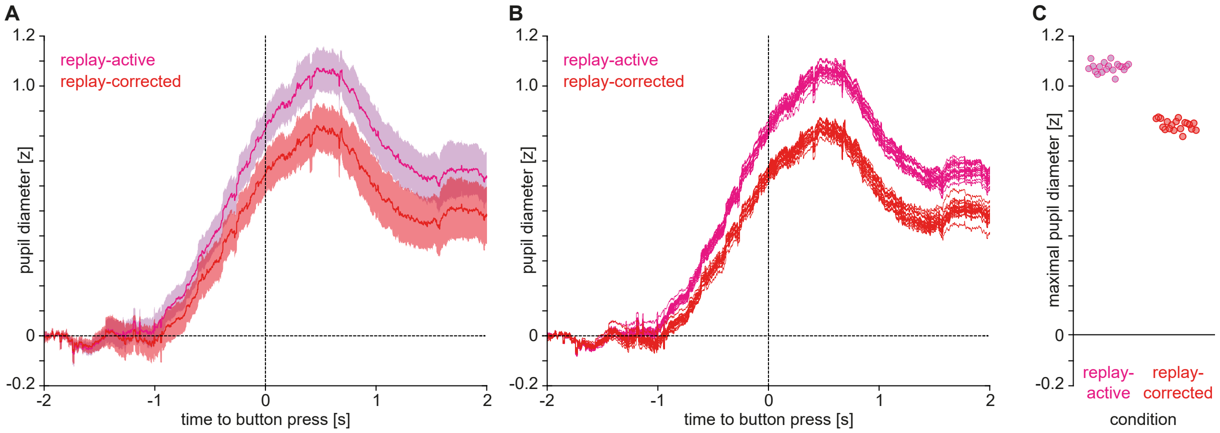

Fig S3. Correction of the replay condition in Study 2. Pupil diameter in the replay-active condition (purple) and after correction by the replay-passive condition (red, 'replay-corrected' as used in the main text). **A**) Pupil diameter between 2 s before and 2 s after the button press, z-normalized and subtractively baseline-corrected to 0 mean between [-2 s and -1.8 s]; solid lines: mean, shaded areas: standard error of mean (sem); red line corresponds to Fig 7 of the main text. **B**) Jackknifed traces (19 per condition), red traces correspond to Fig 8A of the main text. **C**) Maximal amplitudes of the jackknifed traces, red data points correspond to Fig 8B of the main text.

We compared the replay-active and replay-corrected pupil traces in terms of their maximal amplitude as extracted via jackknifing (Fig S3B-C). This revealed a higher amplitude in the replay-active (M = 1.07, SD = 0.020) than in the replay-corrected condition (M = 0.84, SD = 0.021). This difference was significant as confirmed by a jackknifing-corrected t-test ($t_{corrected}(18) = 3.93$, $p < .001$). This difference between replay-active and replay-corrected shows that physical stimulus changes produce a non-negligible enhancement of the pupil dilation amplitude. Consequently, the correction applied in Study 2 for the analysis reported throughout the main text is of relevance to render the replay-condition an adequate comparison for the multistability condition, where there is no physical stimulus change.

Supplement S4: Surrogate analysis

The key finding of the two studies reported here pertains to pupil dilations around the time point of button presses. It is important to make sure that these pupil dilations do not reflect statistical artifacts, such as regular patterns in the underlying pupil trace that are linked to regular button-press patterns. This was tested by surrogate data generation, for which we randomly shuffled the button presses within each block. The time point of the first sound acted as the reference point. The number and duration of button presses was kept, but their order was shuffled, and pupil diameter traces were cut out relative to these surrogate button-press times. The traces were then processed exactly like the actual pupil traces (see main text). The shuffling procedure was repeated 100 times for each condition. This surrogate analysis shows that the pupil dilation effect is abolished when randomly re-positioning the button presses (Fig S4). This visual impression was quantified by examining whether any of the 100 surrogate traces contained samples whose pupil amplitude significantly differed from zero after FDR correction. The maximum number of traces in any condition with at least one significant sample was 4 out of 100, which can be regarded as spurious. Hence, we can rule out the possibility that statistical artifacts were underlying the pupil effects reported in the main text.

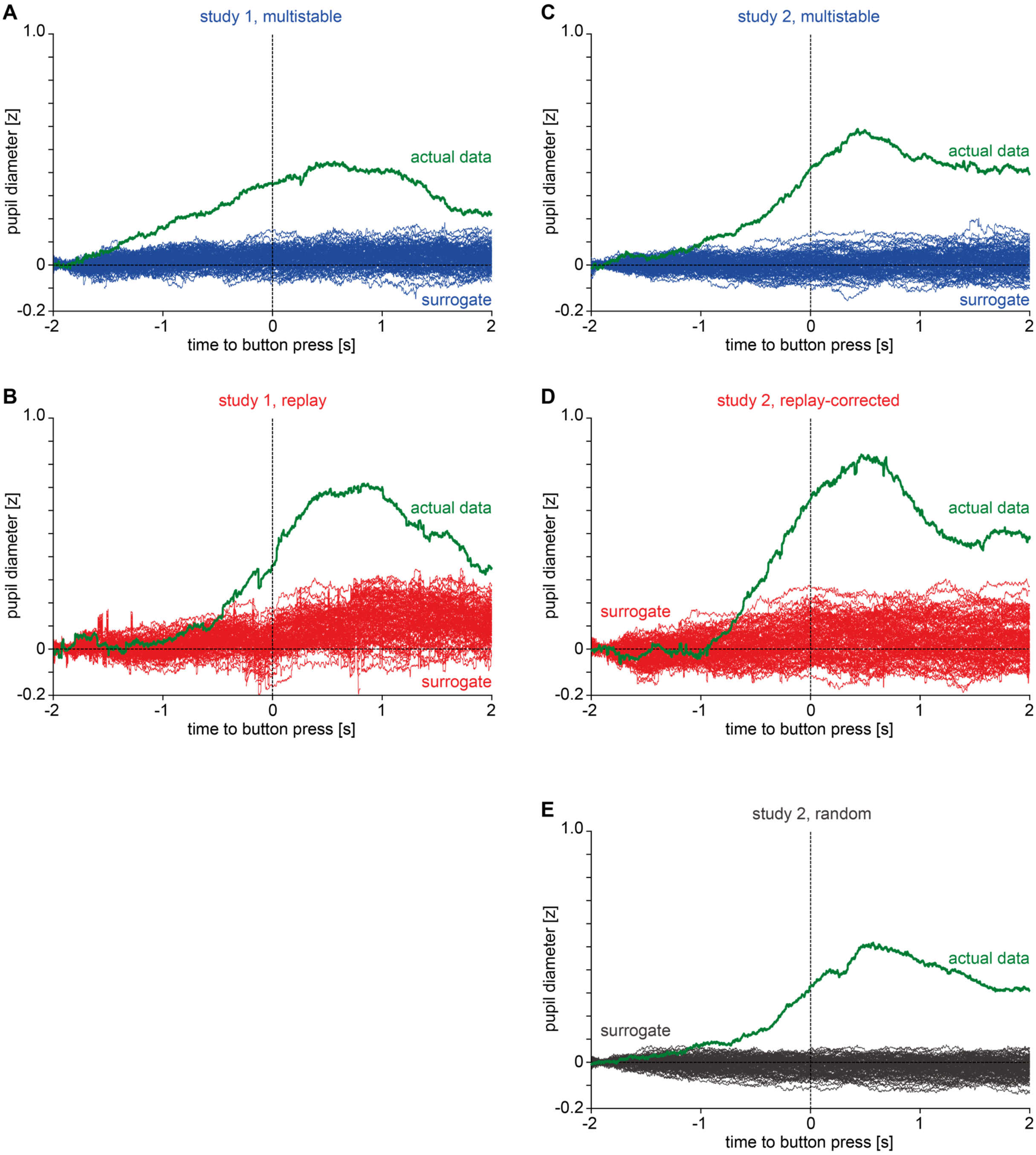

Fig S4. Surrogate analysis. Surrogate traces for the pupil diameter data between 2 s before and 2 s after the button press, separately for each condition in Study 1 (left column, panels A and B) and Study 2 (right column, panels C through E). In each condition, the actual pupil effects (green traces, replotted from Figs 4 and 7 of the main text) by far exceed the surrogate pupil effects (blue/red/black traces). This is true for every single one of the 100 surrogate traces.

Supplement S5: Event-based analysis

To disentangle the magnitude (size) of pupil dilation from the frequency and time course of pupil dilation events, we performed an additional event-based analysis. Following a recent suggestion [2,3], we defined discrete pupil-dilation events (Fig S5A): First, we z-scored the pupil diameter data of each block and filtered it with a 601 ms wide (± 300 ms) boxcar filter; that is, we computed the moving average over 601 consecutive samples and assigned it to the center sample. Missing data (blinks or saccades) were ignored for averaging, the moving average was computed over the non-missing samples in each 601-ms period. For each time point, the slope of the resulting smoothed trace was computed by fitting the optimal linear function to a ± 300 ms time window. The resulting curve is the first derivative of the pupil diameter. By applying the same procedure on the derivative, we obtained the second derivative. Downward zero crossings of this second derivative correspond to local maxima of the slope. These time points were considered *pupil-dilation events* if the slope was positive and the nearest maximum and minimum of pupil diameter (i.e., zero crossings of the slope) had at least 300 ms temporal distance.

For each participant and condition, we computed the fraction of button-press events (defined as in the main analyses) that were preceded by at least one pupil-dilation event within the one-second interval prior to the button press. We found this fraction to be numerically larger for the replay than for the multistable condition in Study 1, though this difference failed to reach significance ($t(18) = 1.13$, $p = .274$; Fig S5B). In Study 2, the fraction was significantly different between conditions, ($F(2,36) = 34.6$, $p < .001$; Fig S5C), with the replay-active condition different from multistable and random condition (both $t(18) > 6.38$, both $p < .001$) and those two conditions statistically indistinguishable from each other ($t(18) = 0.75$, $p = .463$). This

shows that at least for Study 2, switches are more often accompanied by pupil-dilation events in the replay condition than in the other conditions.

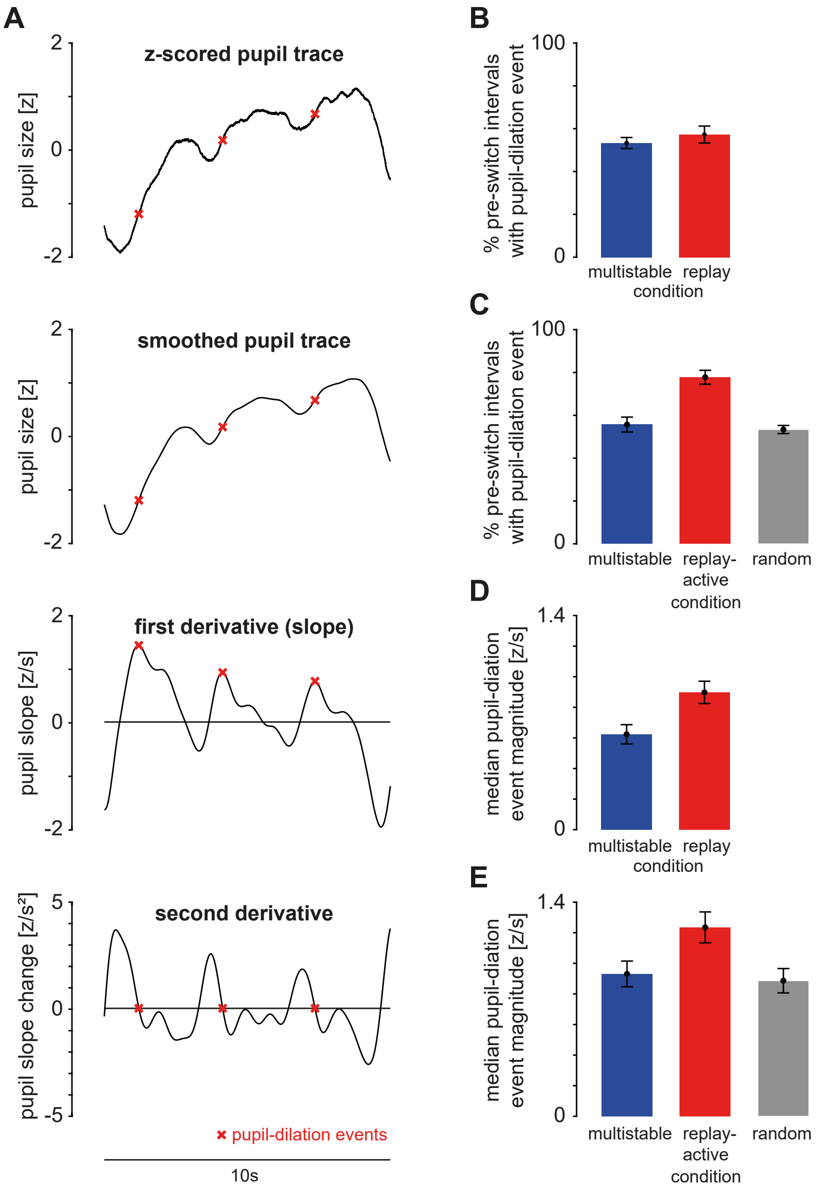

Fig S5. Event-based analysis. A) Method to determine pupil-dilation events illustrated on a 10-s period of raw pupil data: the raw trace (*top*) is filtered (*second row*), the first derivative (*third row*) and the second derivative (*fourth row*) are computed; downward zero-crossings of the second derivative are counted as pupil-dilation events if additional criteria (see text) are met; pupil-dilation events are marked by red crosses; **B, C**) percentage of button presses preceded by a pupil-dilation event in a 1-second interval; **B**) Study 1, **C**) Study 2; **D, E**) median slope of pupil-dilation events in 1-second pre-button interval **D**) Study 1, **E**) Study 2; all error bars denote standard error of the mean across participants.

For the pupil-dilation events within one second before the button-press event, we also computed their median magnitude (i.e., the magnitude of the slope at the time of a pupil-dilation event). For Study 1, we found this magnitude to be larger for the replay than for the multistable condition ($t(18) = 3.73$, $p = .002$; Fig S5D). For Study 2, this magnitude depended on the condition ($F(2,36) = 11.81$, $p < .001$; Fig S5E): the magnitude was larger in the replay-active than in the other two conditions (both $t(18) > 3.49$, both $p < .003$) with no significant difference between random and multistable condition ($t(18) = 0.76$, $p = .457$).

We conclude that the differences in average pupil dilation seen in the main analysis results – at least in Study 2 – stem from two factors: a larger likelihood of a pupil-dilation event occurring around a switch *and* a larger pupil response. Whether the lower likelihood for multistable and random conditions results from the events spreading out over more than the one-second interval or from the weaker response leaving some events undetected remains an open issue. In either case, the event-based analysis corroborates the main analysis: switches in the replay conditions are accompanied by more and stronger pupil-dilation events than in the other conditions.

References

1. van Dam LCJ, van Ee R. The role of (micro)saccades and blinks in perceptual bi-stability from slant rivalry. Vision Res. 2005;45: 2417–2435. doi:10.1016/j.visres.2005.03.013
2. Joshi S, Li Y, Kalwani RM, Gold JI. Relationships between pupil diameter and neuronal activity in the locus coeruleus, colliculi, and cingulate cortex. Neuron. 2016;89: 221–234. doi:10.1016/j.neuron.2015.11.028
3. Zhao S, Chait M, Dick F, Dayan P, Furukawa S, Liao H-I. Pupil-linked phasic arousal evoked by violation but not emergence of regularity within rapid sound sequences. Nat Commun. 2019;10: 1–16. doi:10.1038/s41467-019-12048-1
